# Simian Immunodeficiency Virus Infection of Chimpanzees (*Pan troglodytes*) Shares Features of Both Pathogenic and Non-pathogenic Lentiviral Infections

**DOI:** 10.1371/journal.ppat.1005146

**Published:** 2015-09-11

**Authors:** Edward J. D. Greenwood, Fabian Schmidt, Ivanela Kondova, Henk Niphuis, Vida L. Hodara, Leah Clissold, Kirsten McLay, Bernadette Guerra, Sharon Redrobe, Luis D. Giavedoni, Robert E. Lanford, Krishna K. Murthy, François Rouet, Jonathan L. Heeney

**Affiliations:** 1 Department of Veterinary Medicine, University of Cambridge, Cambridge, United Kingdom; 2 Division of Pathology and Microbiology, Biomedical Primate Research Centre, Rijswijk, The Netherlands; 3 Department of Virology, Biomedical Primate Research Centre, Rijswijk, The Netherlands; 4 Department of Virology and Immunology, Texas Biomedical Research Institute, San Antonio, Texas, United States of America; 5 Southwest National Primate Research Center, San Antonio, Texas, United States of America; 6 The Genome Analysis Centre (TGAC), Norwich, United Kingdom; 7 Twycross Zoo - East Midland Zoological Society, Atherstone, United Kingdom; 8 Laboratoire de Rétrovirologie, Centre International de Recherches Médicales de Franceville, Franceville, Gabon; Emory University, UNITED STATES

## Abstract

The virus-host relationship in simian immunodeficiency virus (SIV) infected chimpanzees is thought to be different from that found in other SIV infected African primates. However, studies of captive SIVcpz infected chimpanzees are limited. Previously, the natural SIVcpz infection of one chimpanzee, and the experimental infection of six chimpanzees was reported, with limited follow-up. Here, we present a long-term study of these seven animals, with a retrospective re-examination of the early stages of infection. The only clinical signs consistent with AIDS or AIDS associated disease was thrombocytopenia in two cases, associated with the development of anti-platelet antibodies. However, compared to uninfected and HIV-1 infected animals, SIVcpz infected animals had significantly lower levels of peripheral blood CD4+ T-cells. Despite this, levels of T-cell activation in chronic infection were not significantly elevated. In addition, while plasma levels of β2 microglobulin, neopterin and soluble TNF-related apoptosis inducing ligand (sTRAIL) were elevated in acute infection, these markers returned to near-normal levels in chronic infection, reminiscent of immune activation patterns in ‘natural host’ species. Furthermore, plasma soluble CD14 was not elevated in chronic infection. However, examination of the secondary lymphoid environment revealed persistent changes to the lymphoid structure, including follicular hyperplasia in SIVcpz infected animals. In addition, both SIV and HIV-1 infected chimpanzees showed increased levels of deposition of collagen and increased levels of Mx1 expression in the T-cell zones of the lymph node. The outcome of SIVcpz infection of captive chimpanzees therefore shares features of both non-pathogenic and pathogenic lentivirus infections.

## Author Summary

The HIV-1/AIDS pandemic is the result of cross-species transmission of simian immunodeficiency virus (SIVcpz) from chimpanzees to humans. Many African primates are infected with SIV, but those studied in captivity generally do not develop disease. However, wild chimpanzees infected with SIVcpz are at increased risk of death and may develop an AIDS-like disease. It has therefore been suggested that the viral features which SIVcpz and HIV-1 share, that differentiate them from other species’ SIV, may be critical in the development of disease in both humans and chimpanzees. Here, we present a long-term follow-up of 7 SIVcpz infected chimpanzees, housed in primate centres in the US and Europe, under similar conditions to other studied models. These animals did not develop an AIDS-like disease, after up to 25 years of infection, and showed features similar to other species where disease rarely develops, such as limited immune activation in the blood. However, they also had significantly reduced CD4+ T-cells and disruption to the secondary lymphoid tissues, normally associated with pathogenic primate lentiviral infections. Thus, while SIVcpz infection of chimpanzees shares features of both pathogenic and non-pathogenic infections, disease has not developed in captivity.

## Introduction

Over 40 different African primate species are naturally infected with a species-specific simian immunodeficiency virus (SIV) [1]. To date, studies into the outcome of SIV infection have been limited to only a few species, in particular African green monkeys (*Chlorocebus genus*) and sooty mangabeys (*Cercocebus atys*) studied primarily in U.S and European primate centres. Such studies have demonstrated the apparent convergent evolution of multiple mechanisms to prevent the development of SIV induced disease in these ‘natural hosts’ of SIV (reviewed [2,3]), and cases of AIDS are extremely rare [4]. A key feature of non-pathogenic SIV infection of African primates is the lack of chronic immune activation, and subsequent destruction of the secondary lymphoid environment that occur in HIV-1 infection of humans.

The outcome of SIV infection of chimpanzees is less well understood than in other African primates. Improving this knowledge is of particular relevance to our understanding of HIV-1 infection, as HIV-1 is the result of a cross-species transmission event of SIV from chimpanzees (of the Central subspecies, *Pan troglodytes troglodytes*) [5]. Also, SIVcpz shares specific viral features with HIV-1 that are not found in the other SIV infections studied [6]. In a study of wild, Eastern chimpanzees (*Pan troglodytes schweinfurthii*), in the Gombe National Park, Tanzania, SIVcpz infected animals had a greater risk of mortality, with evidence of CD4+ T cell depletion, indirect evidence of immune activation, and the development of an AIDS like disease in one animal [7]. These findings are radically different from the outcome of SIV infection in African green monkeys [8,9] and sooty mangabeys [10], studied in primate research centres, and thus it has been suggested that the virus-host relationship between SIVcpz and the chimpanzee differs from that found in other species. It has been proposed that this is either due to a more recent acquisition of SIV in chimpanzees, or due to the presence of viral features of SIVcpz, not found in other SIVs (including the presence of the *vpu* gene and the failure of the SIVcpz *nef* gene to down-regulate the T-cell receptor [11]).

However, it cannot be excluded that the different study environments (wild chimpanzees compared with captive sooty mangabeys) may bias the outcomes of SIV infection of these species. Parasite infections were found to be a major cause of morbidity and mortality in the Gombe chimpanzee cohort [7,12], and the apparent cause of death in one animal thought to be suffering from an AIDS like condition [7]. The other known causes of mortality in the SIV infected chimpanzees were intra-group aggression and other trauma. All these causes of death are less likely in captive primate populations, due to standard veterinary care and behaviour management.

Prior to this landmark study of wild chimpanzees, limited studies of two SIVcpz infected animals facilitated the assumption that chimpanzees were resistant to AIDS following SIVcpz infection. These animals were ‘Marilyn’ and ‘Ch-No’, housed in the USA and The Netherlands respectively. SIVcpz infection of Marilyn (subspecies *troglodytes*) was detected retrospectively, after the death of the animal [13,14]. Marilyn was wild caught, and given the lack of other SIVcpz infected animals in the centre, is presumed to have been infected prior to capture in 1963 at age of approximately 4 years. This animal died in 1985, and was therefore infected for 22 to 26 years without the development of AIDS. The cause of death was apparently due to complications relating to the birth of stillborn twins. Examination of tissues post-mortem revealed only minor disruptions to the secondary lymphoid tissues (plasmacystosis and some degeneration of germinal centres), not consistent with the extensive changes seen in HIV-1 infected humans [13].

Ch-No (‘Noah’, subspecies *schweinfurthii*) is also a wild-born animal infected prior to age 2.5 years [15]. Limited data on this animal, in addition to the description of six chimpanzees (three *schweinfurthii* and three *verus* subspecies) has previously been reported [16]. In that study, blood from Ch-No was used to infect a second animal (Ch-Ni), and blood cells and/or plasma from this animal, obtained two weeks post infection, were used to infect the subsequent 5 chimpanzees. Here we present a long term follow up study of Ch-No, and the 6 experimentally infected animals, for 12 to 25 years post infection, in addition to retrospective analysis of samples obtained throughout infection to characterise the outcome of SIVcpz infection in these captive chimpanzees.

## Results

### Status of the SIVcpz infected cohort

As summarised in Table 1, of seven animals available to study four remained alive and in general good health, as of January 2013, with the time from infection to this date ranging from 15.7 years to 26 years. Three of the animals have died since infection—both of the experimentally infected *schweinfurthii* animals, Ch-Ni and X062, and one of the *verus* animals, X310. In all cases, the resident pathologist determined that the cause of death was cardiac disease, and in no case could the deaths be associated with the development of infectious or neoplastic disease that might be expected as a result of a pathogenic lentivirus infection. Most recent peripheral blood absolute CD4+ T-cell count, viral load and platelet count are also presented in Table 1. Longitudinal data for viral load and absolute CD4+ T-cell count kinetics are shown in S1–S3 Figs. Notably, at the last available time for which CD4+ count data was available, three animals had counts between 200 and 500 cells/μl, which defines stage 2 of CD4+ T-cell loss in HIV-1 infected humans in the Centre for Disease Control (CDC) classification, while one animal had a count below 200 cells/μl, placing it in stage 3. Thrombocytopenia (defined by a platelet count of <50 x10^9^/l) was observed in two animals, categorizing them as CDC clinical stage B.

**Table 1:** Characteristics of the SIVcpz infected cohort.

ID	Sub-species^(1)^	Infection route^(2)^	Age at SIVcpz infection (yr)	Age at death (yr)	Age in 2013 (yr)	Time from infection to death (yr)	Time from infection to 2013 (yr)	Prior HIV infection ^(4)^	Cause of death	Absolute CD4+ count		Viral load		Platelet count		CDC class ^(7)^
										Count (cells/μl)	Time p.i (yr)^(5)^	Viral load (log copies/ml)	Time p.i (yr)	Platelet count (10^9^/l)	Time p.i (yr)	
Ch-No	S	Natural, presumed vertical	0–2.5 ^(3)^	-	26	-	26.0	No	-	402	25	4.4	24	7	24	B2
Ch-Ni	*S*	I.V.	8	18	-	9.8	-	No	Congestive heart failure due to dialative cardiomyopathy	276	9.8†	3.1	9.8†	143	9.8†	A2
X062	*S*	I.V.	19	22	-	3.3	-	IIIb	Cardiomyopathy	1003	1	4.6	0.8	ND^(6)^	ND	A1
X310	*V*	I.V	27	33	-	6.7	-	IIIb	Acute myocardial necrosis	1615	1	<2.4	5	ND	ND	A1
X176	*V*	I.R.	17	-	33	-	16.6	IIIb-p	-	115	14.4	<2.4	14.4	52	14.4	A3
X115	*V*	I.R	19	-	36	-	16.6	SF2	-	229	13.7	2.4	13.7	37	13.7	B2
x284	*V*	I.Vag	12	-	27	-	15.7	No	-	1606	12.3	<2.4	12.3	219	12.3	A1

Relevant details of the SIVcpz infected animals included in this study.

^(1)^ Subspecies of the animal; *schweinfurthii* (*S*) or *verus* (*V*).

^(2)^ Experimentally infected animals (all but Ch-No) were exposed through the intra-venous (I.V.), intra rectal (I.R) or intra-vaginal (I.Vag.) route.

^(3)^ The age of animals Ch-No and Ch-Ni was estimated to be 2.5 years in 1989, at which point Ch-No was already SIVcpz positive. For the calculation of subsequent time intervals Ch-No is assumed to have been infected at birth (age 0).

^(4)^ Some animals were infected with HIV-1 prior to SIVcpz infection. HIV-1 isolates used were IIIb, SF2, or IIIb previously passaged in another chimpanzee (IIIb-p).

^(5)^ The values given for CD4+ T-cell count, viral load and platelet count are the most recent data available. The time of this data varies depending on the animal and type of data, thus the time post SIVcpz infection is given for each value.

^(6)^ No post infection platelet counts are available for X062 and X310.

^(7)^ CDC classification based on CD4+ T-cell count and presence of thrombocytopenia.

^†^ Indicates values obtained from samples taken at the date of death.

To put these results into context, data from uninfected chimpanzees and HIV-infected chimpanzees that have not progressed to disease (the most common phenotype in HIV-1 infected chimpanzees, termed here as HIV non-progressors, HIV-NP), from the centres at which these animals were housed were collated. Details of the animals in each analysis are supplied in the supplementary tables, as this varies between analysis, depending on the availability of samples or data.

**Fig 1:**
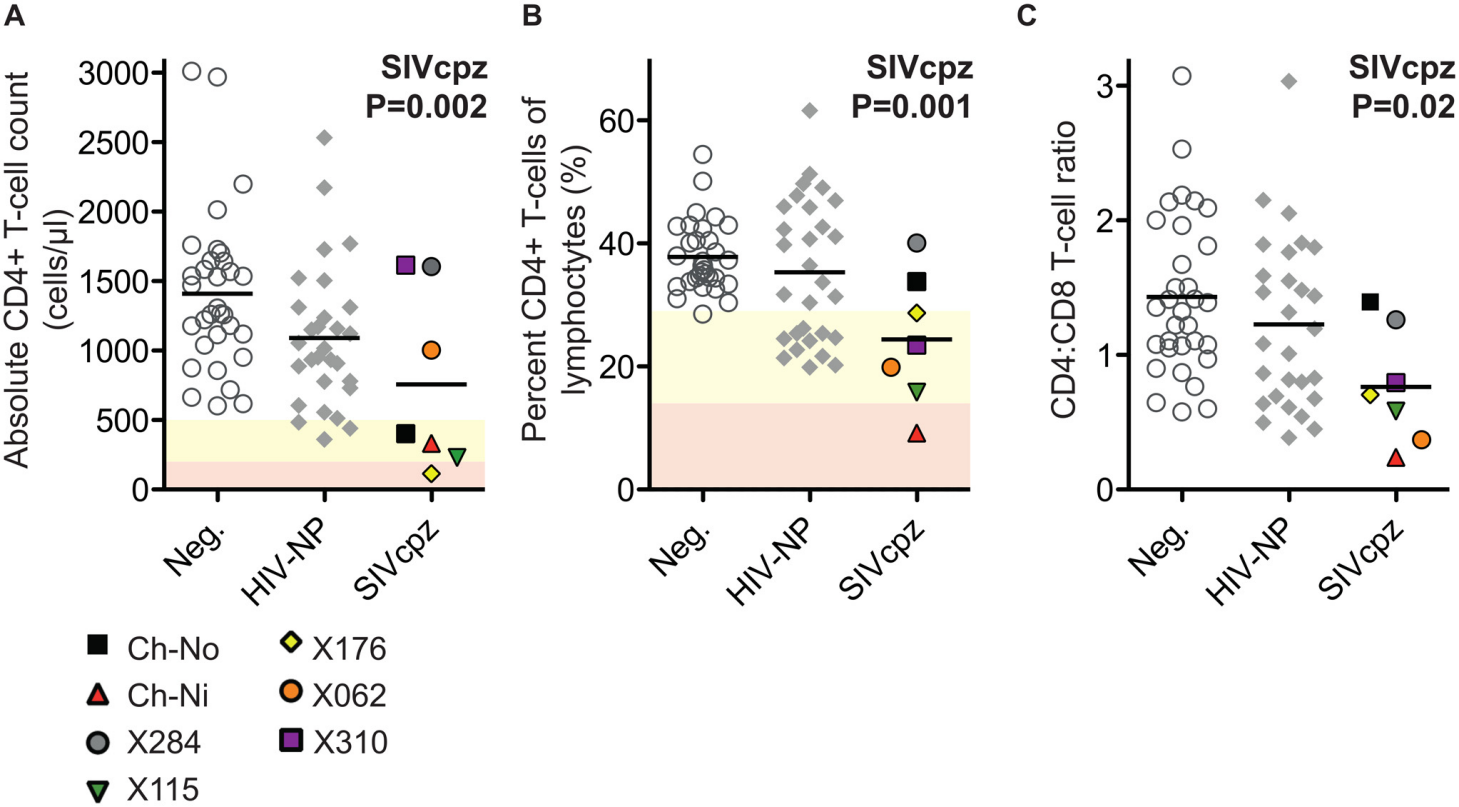
Comparison of CD4+ T-cell levels in uninfected, HIV-1 infected and SIVcpz infected animals. Levels of peripheral blood CD4+ T-cells were quantified by flow cytometry in chimpanzees that were uninfected (Neg., n = 32), HIV-1 non-progressors (HIV-NP, n = 29), or SIVcpz infected (SIVcpz, n = 7). Levels were quantified as (A) Absolute CD4+ T-cell counts, (B) percentage CD4+ T-cells of the lymphocyte population, and (C) the CD4:CD8 T-cell ratios. Shading corresponds to CDC categorisation for CD4+ T-cell levels (absolute count and percentage of lymphocytes) in HIV-1 infected humans, where yellow indicates stage two and red indicates stage one. P value shown indicates that SIVcpz infection was a significant predictor of reduced CD4+ T-cell levels, in a general linear model including age, gender, and HIV-1 and SIVcpz status. Full details of analysis are available in S1–S3 Tables. Each SIVcpz infected animal is represented by a unique symbol, as indicated in the legend. Horizontal black bars indicate the mean value in each group. Data shown for the SIVcpz infected animals are the most recent available, detailed in Table 1 as the time post infection for absolute T-cell count.

The absolute CD4+ T-cell count, the percentage that CD3+CD4+ cells make of the total lymphocyte population, and the CD4:CD8 ratio of these animals were compared with uninfected and HIV infected controls. SIV infection was found to have a significant negative impact on all three of these parameters (Fig 1) in a general linear model, with age, gender and HIV-1 infection status as additional covariates (detailed in S2 Table).

### Peripheral blood T-cell activation in SIVcpz infected chimpanzees

**Fig 2:**
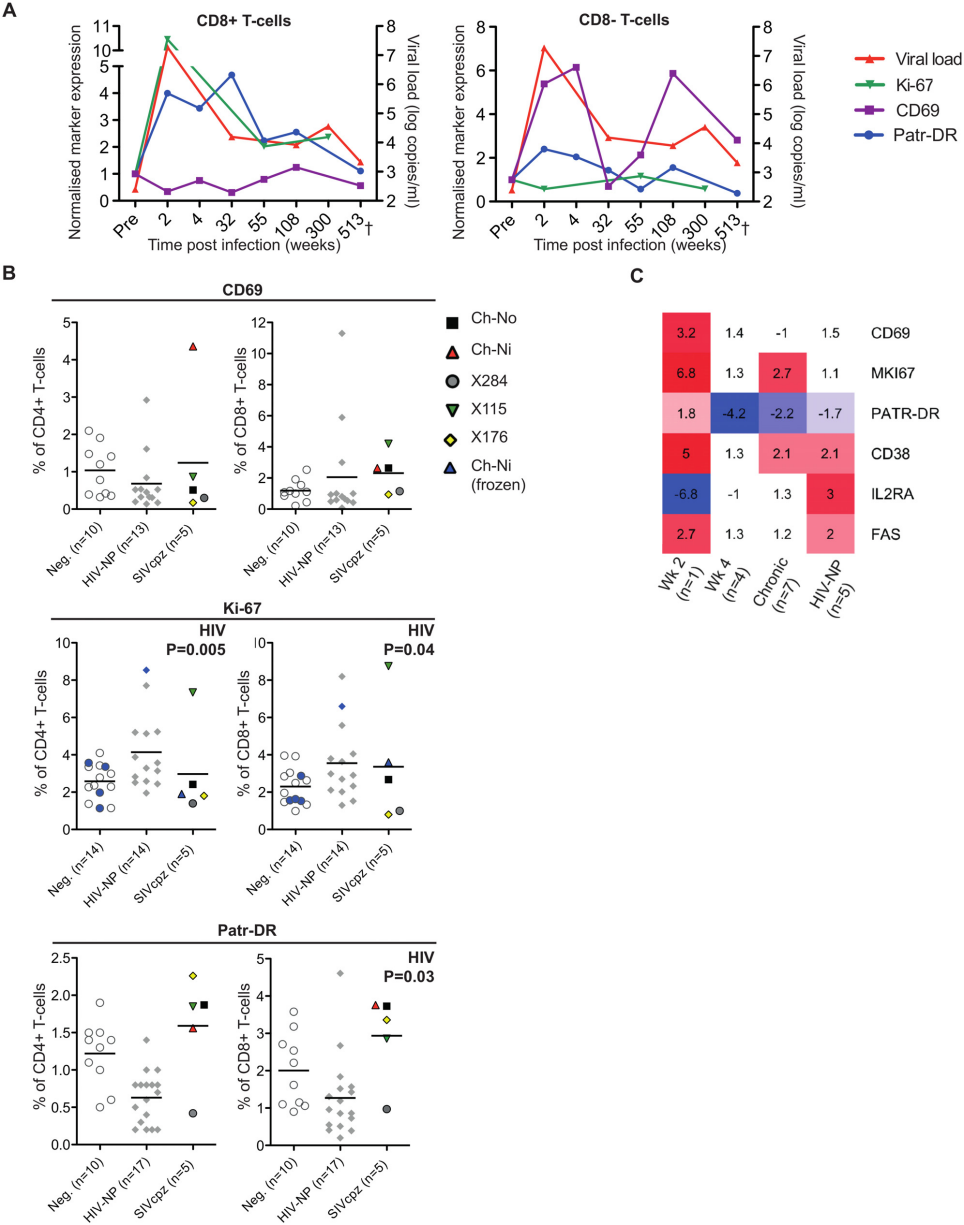
Peripheral blood T-cell activation in SIVcpz infected animals. (A) Longitudinal measurements of T-cell activation in the CD8+ and CD8- T-cell populations of the animal Ch-Ni. Each marker was normalised to pre-infection values: data is expressed as fold change compared to this. Viral load is shown for reference. Ki-67 measurements were carried out on cryopreserved cells. (B) T-cell activation markers measured in uninfected, HIV-1 and SIVcpz infected chimpanzees in the CD4+ compartment and CD8+ compartment. Ki-67 measurements established from cryopreserved cells are coloured blue. Horizontal black bars indicate the mean value in each group. Data was unavailable for X310 and X062. P-value shown indicates that HIV-1 status was a significant predictor of the plotted variable in a general linear model including age, gender, and HIV-1 and SIVcpz status. Data shown for the SIVcpz infected animals is the most recent available, detailed in Table 1 as the time post infection for last absolute T-cell count, with the exception of measurements of Ki-67 in cryopreserved samples from Ch-Ni, which were week 300 p.i. (C) Heatmap showing expression levels of the indicated genes in RNA-seq transcriptomics analysis of peripheral blood mononuclear cells (PBMCs). Data is shown as fold upregulation compared to a group of uninfected animals n = 3. Full information for the samples used is provided in S7 Table.

Markers of T-cell activation were measured during the acute and chronic phase of infection for one of the experimentally infected animals (Ch-Ni), shown in Fig 2A. As Ki-67 was not measured contemporaneously, cryopreserved samples were used to measure this marker, with samples from pre-infection, and at week 2, 55 and 300 post infection (p.i.). In addition as marker expression was not measured contemporaneously on CD4+ T-cells at some time points, longitudinal data for Ch-Ni is displayed for T-cells that are CD8 positive or CD8 negative. Both Patr-DR (MHC class II), and Ki-67 were upregulated in the CD8+ T-cell population at week two p.i., with reduced expression of these markers by one year p.i., albeit at levels that remain higher than pre-infection values. Only CD69 was up-regulated on the CD8- population, with fluctuating values throughout chronic infection.

Data from the chronic phase of infection was examined for all five SIV infected animals for which flow cytometric analysis was carried out. When it was compared to uninfected and HIV-1 infected animals, the effect of SIVcpz infection on these markers appeared to be small or not apparent (Fig 2B, details of groups and statistical analysis S3 & S4 Tables). Non-progressive HIV-1 infection was associated with small but significant increases in Ki-67 expression in both the CD4+ and CD8+ T-cell populations, while SIVcpz appeared to induce a similar or smaller (and not significant) effect. Curiously, HIV-1 infection was associated with a small but significant suppression of Patr-DR (MHC-II) expression on CD8+ T-cells. Transcriptomic analysis of cryopreserved peripheral blood mononuclear cells (PBMCs) was also carried out on a subset of samples, (samples used are detailed in S5 Table). Fig 2C shows the transcriptomics data for the T-cell markers studied by flow cytometry, along with some other well studied T-cell activation markers that have been shown to be elevated in HIV-1 infected individuals: *CD38*, *IL2RA* (CD25) and *FAS* (CD95)[17–21]. Transcriptional level data revealed similar patterns as the flow cytometry. Compared with three uninfected animals, the one available sample from week two p.i. showed more than 2-fold increased levels of transcripts for CD69, Ki-67 (MKI67), CD38, and FAS. Of these, none were more than 2 times elevated in both the week 4 post-infection samples (n = 4), though Ki-67 and CD38 were also more than two-fold upregulated compared to control samples in the chronic phase (n = 7) of SIVcpz infection. A more complete analysis of the effect of SIV/HIV-1 infection on genes shown to be upregulated during T-cell activation *in vitro* is shown in S4 Fig. While a number of genes are upregulated or downregulated in all groups compared to uninfected controls, there are a number of T-cell activation associated genes that are only more than two-fold upregulated in the week two sample, such as *IFNG*, *GZMB*,*PRF1*.

### Soluble markers of immune activation

**Fig 3:**
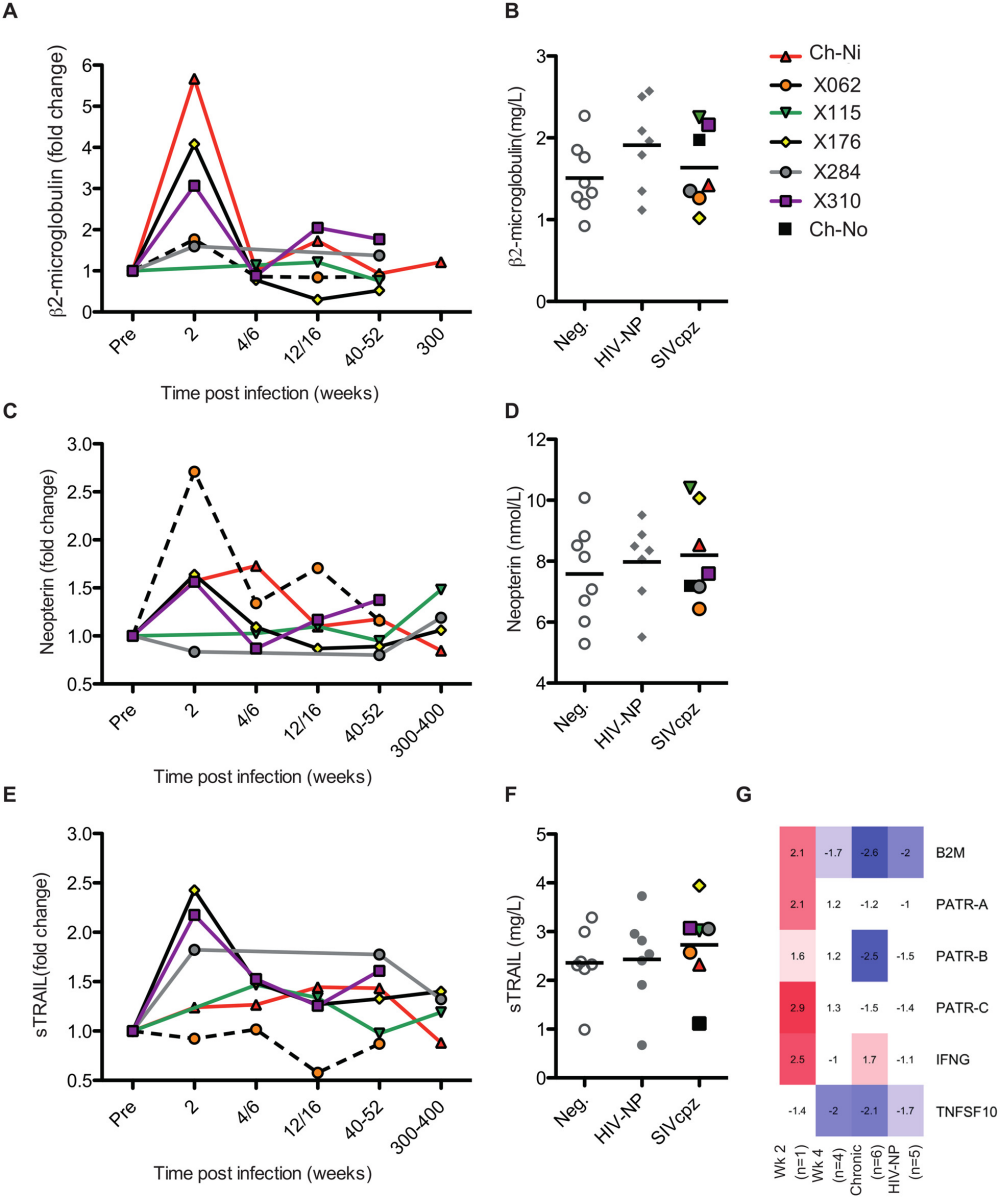
Soluble markers of immune activation in the plasma of SIVcpz infected chimpanzees. (A, C, E) Plasma levels of β2-microglublin, neopterin, and sTRAIL determined by ELISA, during the acute and chronic phase of infection in the experimentally infected animals. Levels of each marker were normalised to the pre-infection level for each animal. Horizontal black bars indicate the mean value in each group. As sampling times and/or sample availability was not identical for each animal, comparable time points are grouped (e.g. week 4 and week 6 are displayed as a single time point). (B, D, F) Levels of each marker in the chronic phase (including Ch-No) of SIVcpz infection compared with values for uninfected and HIV-1 infected chimpanzees, and displayed as absolute values. (G) Heatmap showing expression levels of the indicated genes in RNA-seq transcriptomics analysis of PBMCs.

The soluble markers of immune activation β2 microglobulin (β2M), neopterin, and soluble tumour necrosis factor-alpha-related apoptosis-inducing ligand (sTRAIL) were measured in the plasma of SIVcpz infected animals during acute and chronic infection by ELISA (Fig 3, details of groups in S6 Table). β2M levels were upregulated in all experimentally infected animals at week two p.i., returning to pre-infection levels at week 4 p.i.. When chronic phase β2M levels of all SIVcpz infected animals (including Ch-No) were compared to uninfected or HIV-1 infected animals, there was no evidence for increased expression of this marker in SIVcpz infected animals.

A similar pattern was found for neopterin levels, with the exception that this marker was not elevated at week two in one animal (x284). Comparing samples taken in the chronic phase of infection, there is a slight but not significant trend for SIVcpz infected animals to have increased levels of plasma neopterin. Analysis of sTRAIL produced similar findings, with three animals showing elevated levels of this marker at week two p.i., and with a slight, but not significant, trend for HIV-1 and SIVcpz infected animals to have higher levels in the chronic stage of infection. The transcriptional analysis of genes related to these markers was also investigated (Fig 3G). Similar to the findings by ELISA, transcripts for *B2M* and MHC-class I molecule genes were only elevated in the week two p.i. sample, as were transcripts for interferon-γ, which drives neopterin production. At the transcriptional level, *TNFSF10* (TRAIL) was downregulated in all groups except the week two p.i. sample. However, it must be noted that the transcriptional level data is derived from samples that do not exactly match those for which plasma samples were available, and only represent expression in peripheral blood mononuclear cells (PBMCs) which are not likely to be the sole source of plasma soluble proteins.

### Analysis of structural changes in the secondary lymphoid environment

Limited lymphoid material was available from animals in the SIVcpz infected cohort: necropsy materials from Ch-Ni and X062, and a biopsy from Ch-No, taken at the age of 8 years old, allowing the determination of the effect of chronic SIVcpz infection in a total of three animals. Biopsies were also available from three experimentally infected animals (X176, X115 and Ch-Ni) representing acute (week two) and early (week 12/16) time points of infection. These samples were compared with necropsy materials from uninfected and HIV-1 infected control animals.

**Fig 4:**
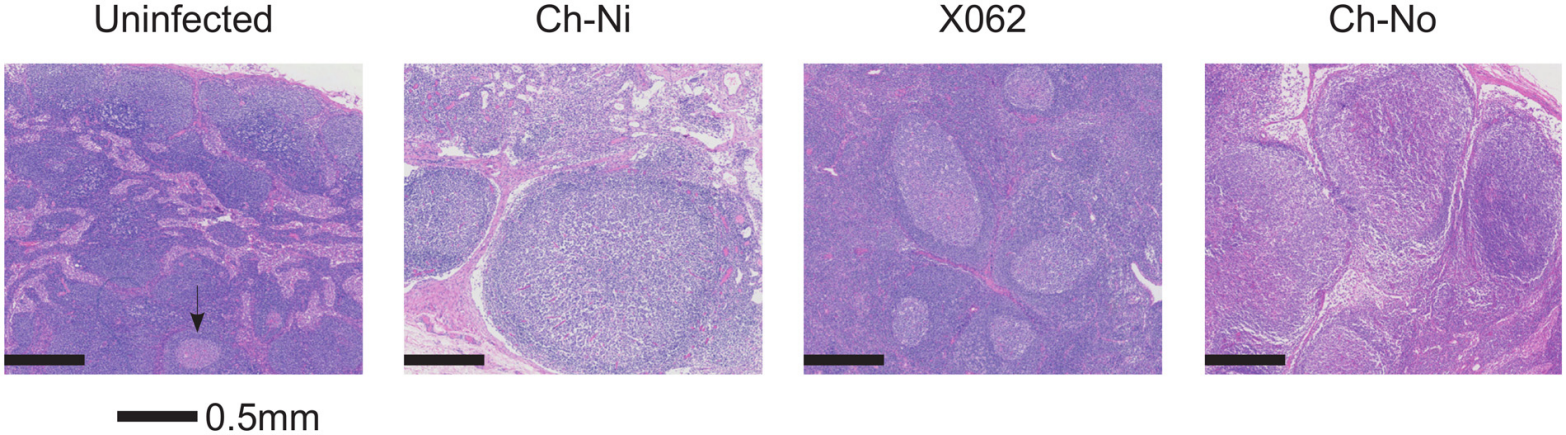
Lymphoid hyperplasia in SIVcpz infected chimpanzees. Representative images from one uninfected chimpanzee, and the three available lymph node samples from chronic SIVcpz infected chimpanzees (detailed in Table 2). Arrow indicates the one normal follicle visible in the image from the uninfected animal.

Pathologist examination of lymph nodes from the three animals in the chronic phase of infection revealed a number of phenomena found in HIV-1 infected patients, including varying degrees of lymphoid hyperplasia in these animals; Ch-Ni (mild to moderate), X062 (marked) and Ch-No (marked). Vascular proliferation was noted in both Ch-No and Ch-Ni (Table 2). Representative images demonstrating the follicular hyperplasia in chronic infected animals are shown in Fig 4. Follicular hyperplasia was not evident in any of the three animals at week two p.i., while in week 12/16 samples, follicular hyperplasia was present in two of the three animals. Follicular hyperplasia was also absent in all 7 uninfected control animals, but present in 2 of 5 lymph node biopsies from HIV-1 infected chimpanzees.

**Table 2:** Pathologist observations of tissues from chronic SIVcpz infected chimpanzees.

ID	Type of material	Time post infection (years)	Histopathology
Ch-No	Biopsy	7.9	• Moderate follicular hyperplasia
			• Mild to moderate fibrosis and altered vascular network (in the corona and paracortex)
Ch-Ni	Necropsy	9.8	• Mild to moderate follicular hyperplasia
			• Moderate follicular and paracortical fibrosis and mildly increased vascularization
X062	Necropsy	3.4	• Moderate follicular and paracortical hyperplasia

Findings of a veterinary pathologist (IK), upon analysis of H&E, Masson's Trichrome and anti-collagen-I stained slides of peripheral lymph nodes.

**Fig 5:**
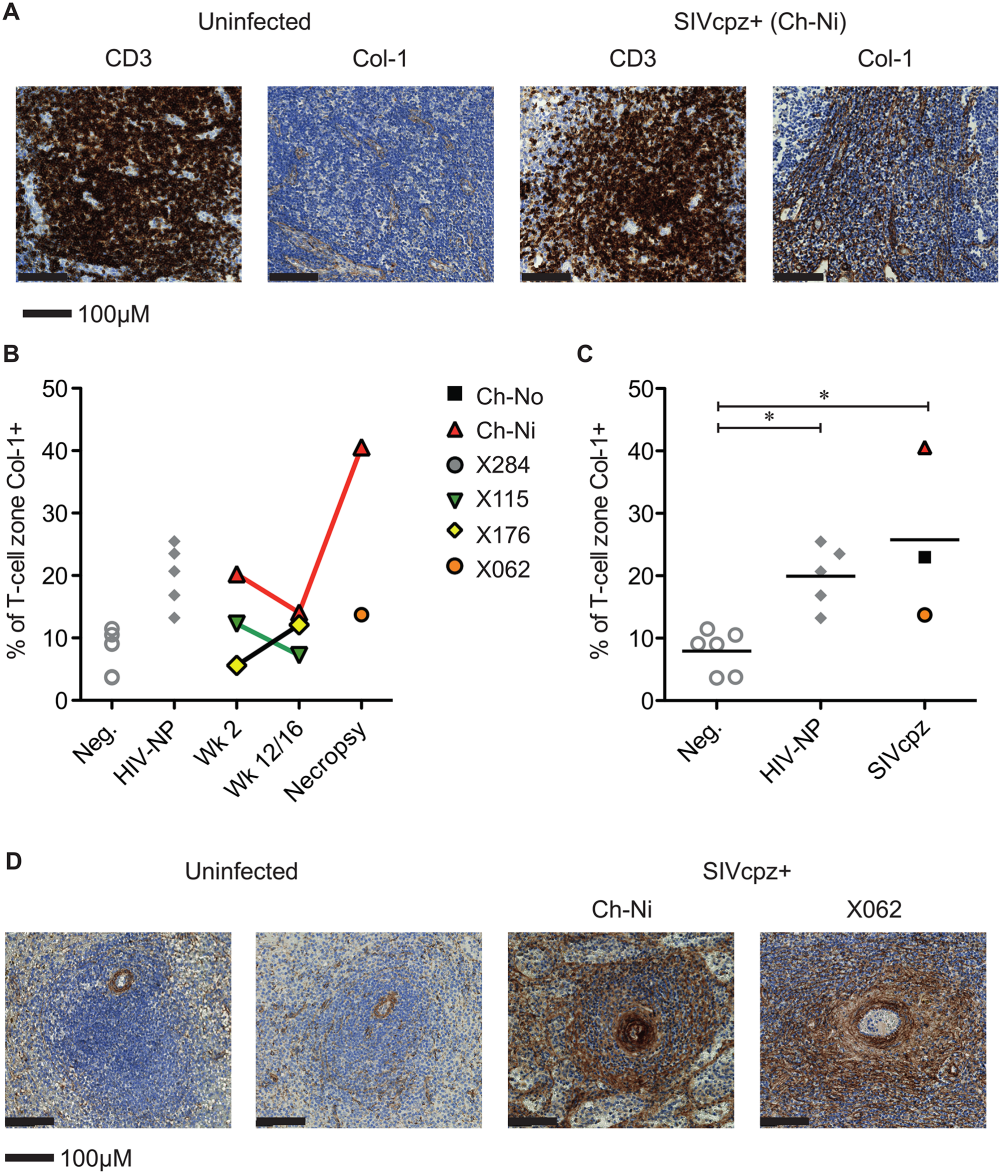
Collagen deposition in SIVcpz infected animals. (A) Representative images of anti- CD3 and anti-collagen 1 immunostainings of lymph nodes from an uninfected chimpanzee and from the chronically SIVcpz infected chimpanzee Ch-Ni. (B) Quantification of collagen I in T-cell zones from experimentally infected animals in early and chronic infection. (C) Comparison of the same parameter in uninfected chimpanzees (n = 6) and chronic HIV-1 (n = 5), and chronic SIVcpz (n = 3) infected chimpanzees. Horizontal black bars indicate the mean value in each group. Group details provided in S7 Table. * indicates P<0.05 in a Kruskal–Wallis Test with Dunn’s post test. (D) Representative images of splenic PALS of two uninfected animals and the two SIVcpz infected animals for which spleen samples were available.

Available tissues were analysed by immunohistochemistry using an anti-collagen 1 antibody and the percentage of T-cell zones staining for collagen-1 was quantified (Fig 5, details of groups in S7 Table). In both chronic HIV-1 and SIVcpz infected animals there was significantly increased levels of collagen deposition in the T-cell zone compared to uninfected animals, with particularly high levels of deposition in the SIVcpz infected animal Ch-Ni. Interestingly, the degree of collagen deposition in the T-cell zones of this animal were at similar levels to those seen in HIV-1 infected animals at only week two p.i. Increased collagen deposition was also observed in the PALS of the spleen of the two deceased SIVcpz infected animals.

### Analysis of immune activation in the peripheral lymphoid tissues

**Fig 6:**
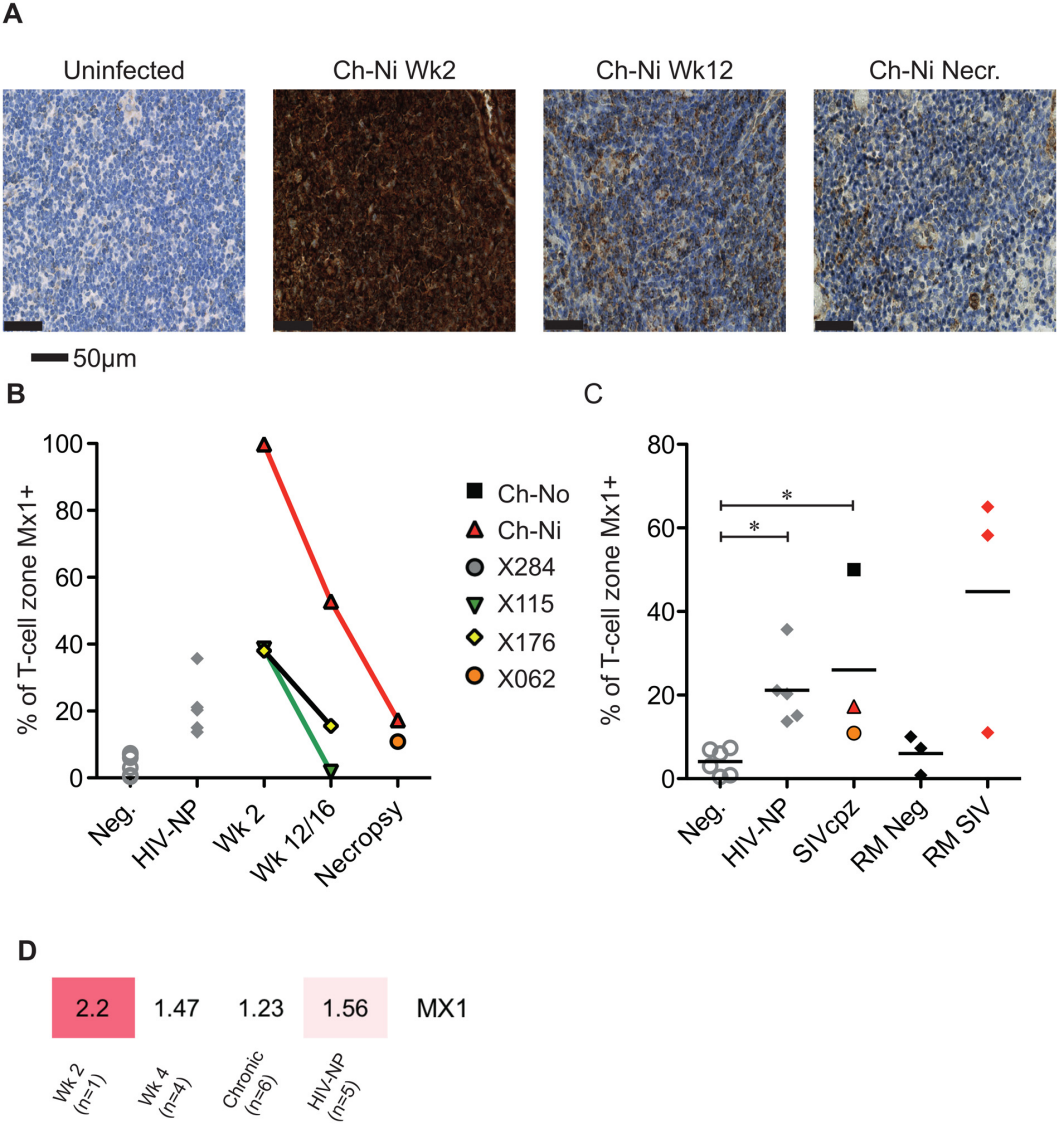
Analysis of Mx1 expression in the lymph nodes of SIVcpz infected chimpanzees. (A) Representative images of anti-Mx1 staining in the T-cell zones of a lymph node of an uninfected chimpanzee, and of Ch-Ni at week 2 and 12 post infection, and at necropsy. (B) Proportion of lymph node T-cell zones staining for anti-Mx1 staining over the course of experimental SIVcpz infection. (C) The same analysis in uninfected chimpanzees (n = 6), chronic HIV-1 infected (n = 5) chronic SIVcpz infected chimpanzees (n = 3), uninfected rhesus macaques (RM neg, n = 3) and SIVmac infected rhesus macaques (RM SIV, n = 3). Horizontal black bars indicate the mean value in each group. * indicates P<0.05 in a Kruskal–Wallis test with Dunn’s post test. (D) Transcription level expression of Mx1 in PBMCs in the RNA-seq cohort, numbers indicate fold upregulation compared to the control group (n = 3).

Progressive HIV and SIV infections are associated with chronic immune activation measurable in the secondary lymphoid tissues. Mx1 (also known as MxA) is an antiviral factor with no activity against HIV-1 [22], but which is upregulated in response to type I interferon stimulation. Persistent expression of Mx1 in the lymph node distinguishes pathogenic and non-pathogenic lentiviral infections [23], as it is rapidly down-regulated after acute infection in African ‘natural host’ species. In the three experimentally infected animals, Mx1 staining was elevated at week two post-infection compared to uninfected controls in the T-cell zone (Fig 6). These levels were reduced by week 12/16, remaining elevated at this time point only in Ch-Ni. In the chronic phase of infection, levels of Mx1 in the T-cell zone was significantly elevated compared to uninfected controls. In particular, the naturally infected animal Ch-No had high levels of Mx1, approaching the levels found in SIVmac infected rhesus macaques, included here to provide additional context. At the transcriptional level, Mx1 expression in PBMCs was only elevated more than two fold in the week two sample. The expression patterns of other known Type I interferon modulated genes is shown in S5 Fig.

Proliferating T-cells in the lymph node were also measured, by quantifying the number of Ki-67 positive cells in the T-cell zone of the lymph node (S6 Fig). There were no significant differences in chronic HIV-1 or SIVcpz infection compared to uninfected animals, though there were highly elevated levels in the animal Ch-Ni in the acute phase of infection.

### Autoimmune anti-platelet antibodies in SIVcpz infected animals

**Fig 7:**
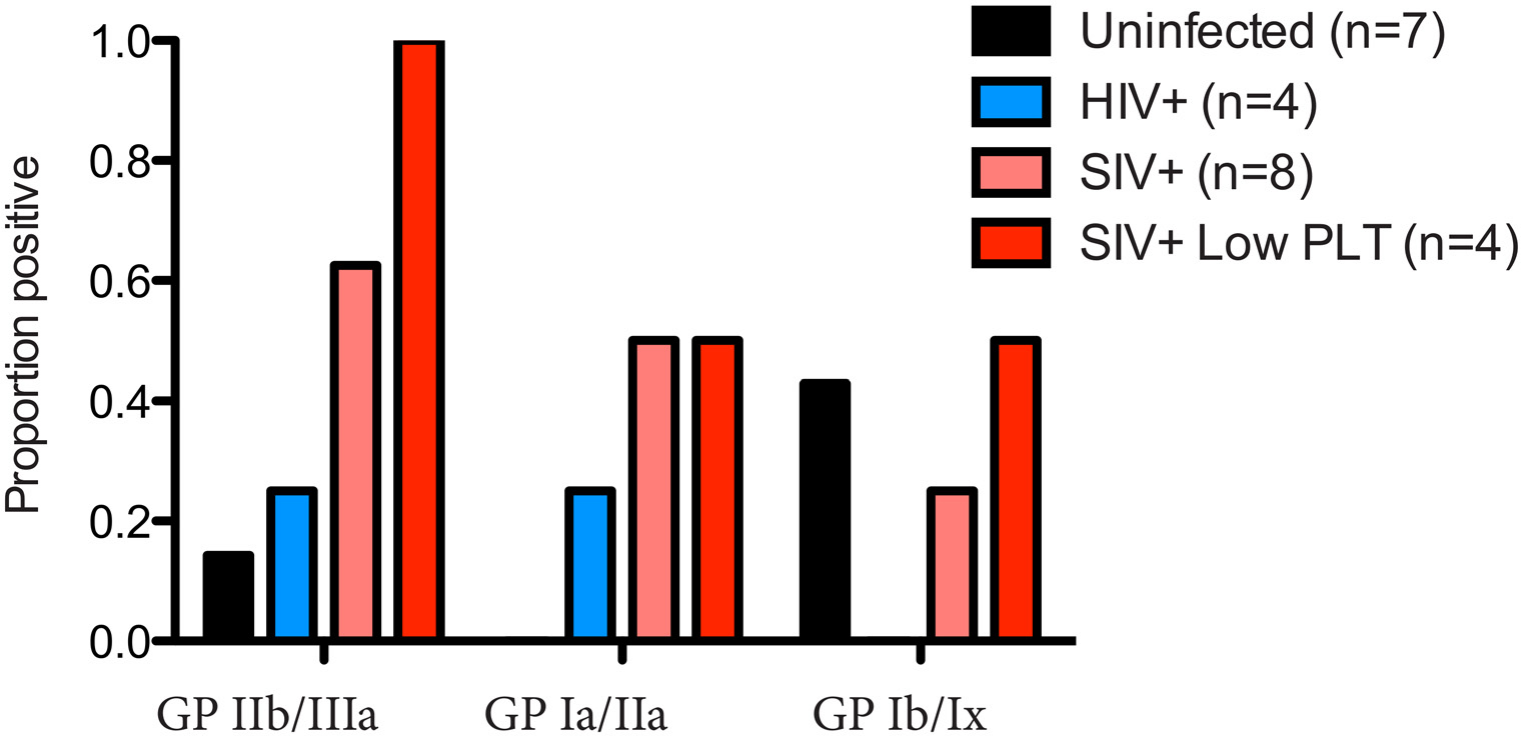
Prevalence of anti-platelet autoimmune antibodies. The presence of anti-platelet antibodies, specific for the glycoprotein (GP) complexes IIb/IIIa, Ia/IIa and Ib/IX was determined in plasma from uninfected, HIV-1 infected and SIVcpz infected animals. The prevalence of these antibodies in the four animals with low platelet counts is also shown.

Chronic thrombocytopenia has previously been reported for Ch-No [16]. In addition, of the five experimentally infected animals with post infection platelet counts available, two had platelet counts falling within the bottom 5th percentile for their age and gender category (5th percentile for adult males = 130.5 x10^9^/l, for adult females = 150.87 x10^9^/l [24]). Chronic thrombocytopenia is also reported for Cam-155, a naturally SIVcpz infected chimpanzee housed in a primate sanctuary in Cameroon with AIDS like clinical signs [25]. Noting that auto-immune antibodies against platelet glycoproteins GP II/IIIa and GP Ib/Ix are associated with thrombocytopenia in SIV infected rhesus macaques [26], the presence of such antibodies was determined in this cohort. A plasma sample from Cam-155 was also available for analysis and included here (this sample was excluded from other analyses as it was exposed to poor storage conditions, but was included here due to the relative stability of antibody molecules, and after confirmation of IgG presence by a Protein A capture ELISA). Thus, the presence of these antibodies were tested in a total of 8 animals (7 described in Table 1, and Cam-155). Most strikingly, antibodies against GP II/IIIa were found in 5 of 8 SIVcpz infected animals, including all four animals determined to have thrombocytopenia or low platelet counts, Fig 7.

### Measurement of soluble CD14

**Fig 8:**
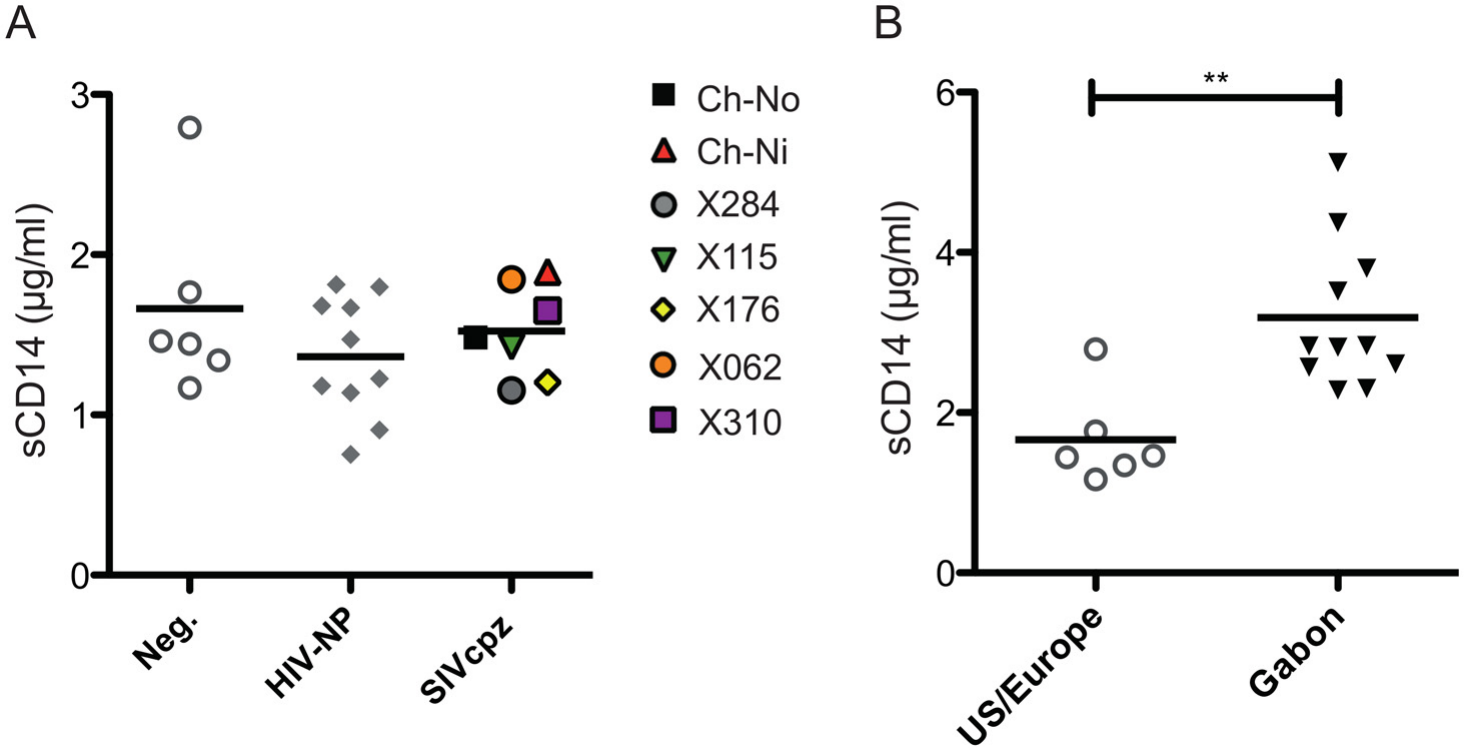
Levels of plasma soluble CD14 in uninfected and SIVcpz infected chimpanzees. (A) Levels of plasma sCD14 were determined in uninfected and chronic HIV-1 and SIVcpz infected chimpanzees. (B) Levels of sCD14 in uninfected chimpanzees held in two primate centres in the US and Europe, compared with chimpanzees housed at the CIRMF, Gabon. Horizontal black bars indicate the mean value in each group. ** indicates a P-value of <0.01 in a Mann-Whitney U test, two tailed.

No significant difference between chronic SIVcpz infected animals and HIV-1 or uninfected animals was found in the concentration of plasma soluble CD14 (Fig 8a). Levels of sCD14 were also compared between uninfected chimpanzees housed in primate research centres in the U.S and Europe, with the levels found in chimpanzees housed at the International Centre of Medical Research, Franceville (CIRMF), Gabon. Notably, levels of sCD14 were significantly greater in animals housed in the African primate centre than in animals housed in the US or Europe (Fig 8b).

## Discussion

The finding that wild chimpanzees of the *schweinfurthii* subspecies in the Gombe national park were at greater risk of mortality when infected with SIVcpz [7] has led to the view that SIVcpz infection of chimpanzees presents a greater risk of a pathogenic outcome than SIV infection of other African primates. This is of particular significance as SIVcpz is the source of HIV-1 in humans, and is one of only a few SIVs to share specific viral features with HIV-1, such as the presence of the *vpu* gene, and the failure of Nef to down-regulate CD3 [11].

We present here the first long-term study of multiple SIVcpz infected chimpanzees, housed in primate centres in the US and Europe, conditions similar to the majority of studies of sooty mangabeys and African green monkeys, upon which our understanding of SIV infection of African ‘natural host’ primate species is based. Most notably, SIVcpz infection in this cohort was not associated with the development of an AIDS like disease. In all of the animals that have died post infection, death was attributed to cardiac disease. While HIV-1 infection of humans is associated with heart disease [27], it should be noted that deaths related to cardiac problems are common in captive chimpanzees, particularly in males [28]. This has been studied in chimpanzees at Texas Biomedical Research Institute (formally Southwest Foundation), where the majority of animals in this cohort were housed. The mean age of death in male chimpanzees dying of heart disease is approximately 26, with deaths from heart disease noted as young as 10 years old at this centre [28]. As such, it is not necessary to assume an effect of SIVcpz infection on the development of heart disease in three animals reported here, which died 18, 22 and 33 years of age.

Despite a lack of AIDS-defining illnesses or deaths in this cohort, SIVcpz infection was associated with significant depletion of CD4+ T-cells. This is consistent with the findings of CD4+ T-cell loss in naturally infected animals in the wild [7], and the two investigated chimpanzees in captivity in primate facilities in Africa [25,29]. However, despite the loss of CD4+ T-cells in SIVcpz infected animals in this study, SIVcpz infection shared features with the non-pathogenic infection of sooty mangabeys and African green monkeys with their species-specific viruses. In particular, SIV infection of natural host species is associated with a rapid reduction in innate and adaptive immune activation after the acute phase, while in pathogenic infection of humans and Asian primates, immune activation persists throughout the chronic phase. In the peripheral blood of the chimpanzees studied here, soluble and cellular markers of immune activation reverted to normal levels shortly after acute infection, as occurs in other well studied natural hosts [9,30]. The mechanisms by which immune activation is brought under control in even the most well studied species remains controversial.

The low viral load at several of the chronic time-points observed here could explain the low levels of immune activation found in this study. However, it should be noted that in most measurements, immune activation was brought rapidly under control after only weeks of infection, despite relatively high viral loads for at least the first year of infection in all animals. Secondly, peripheral blood immune activation was also low in the naturally infected animal, Ch-No, despite relatively high viral loads (4–5 log copies/ml) throughout.

Progressive HIV and SIV infections are also associated with pathological changes to the structure of the secondary lymphoid environment. Interestingly, unlike markers of activation in the peripheral blood, persistent and significant changes were evident in the peripheral lymphoid tissues of SIVcpz infected animals. These included follicular hyperplasia, increased levels of Mx1 expression, and increased deposition of collagen, all of which are features of pathogenic lentiviral infection. Indeed, it is these features that most strongly differentiate the chimpanzees studied here from other SIV infected natural hosts. Some persistent changes, including CD4 loss, increased T-cell activation and an increased levels of transcription of interferon induced genes can be identified through flow cytometric and transcriptional analysis of SIV infected African green monkeys and sooty mangabeys [9,10,30,31]. However, changes in the lymphoid tissues to not appear to be shared with other African primate species. Follicular hyperplasia has never been found to be associated with SIV infection of other natural hosts [32–34], and both collagen deposition and chronic increased Mx1 expression (as measured at the protein level), segregate pathogenic and non-pathogenic infection models [23,35,36].

Thus, combined with the findings of Keele *et al* [7], it would appear that significant changes to the secondary lymphoid tissue structure, including collagen deposition, are features that segregate SIVcpz infection of chimpanzees from the SIV infections of any other African primate, regardless of study setting. It should be noted for future studies that these phenomena occurred here without changes of comparable magnitude in assays of the blood, in terms of the systemic immune activation that accompanies such changes in the lymphoid tissues in other species where this occurs. It is possible that a greater sensitivity to detect changes in immune activation would have been achieved if specific subsets (specifically non-naïve subsets) of CD4+ and CD8+ T-cells were examined, but unfortunately such measurements could not be carried out with samples available., It is also worth noting however that most papers that describe significant T-cell activation in HIV infected humans have generally studied entire CD4 and CD8 populations [17–21,37]

The possibility that SIVcpzANT is a naturally attenuated virus must be considered. That all the animals in this cohort were infected with the same strain of SIVcpz is a major limitation in using this study to draw broader conclusions about the outcome of SIVcpz infection. Unfortunately, it is extremely difficult to determine if SIVcpzANT differs significantly from other SIVcpz isolates. While host factors play a large role in the outcome of HIV-1 infection of humans, naturally occurring attenuated HIV-1 viruses have been isolated and sequenced from long term non-progressor HIV-1 infected patients (reviewed in [38]). Mutations in these viruses which might explain how they are susceptible to host immune response, replication impaired, or in any other way attenuated, only become apparent by comparison with the vast number of HIV-1 sequences available from patients showing a typical course of disease. By contrast, sequences of SIVcpz from *schweinfurthii* subspecies animals are limited to SIVcpzANT, closely related sequences from the Gombe cohort in Tanzania (SIVcpzTAN), and only two additional sequences (a full-length genome from an animal in the DRC SIVcpzBF1167, and a partial genome from an animal in Uganda, SIVcpzUG31. Without additional, more closely related sequences for comparison, in addition to known outcomes of infection, there is no readily apparent methodology to determine if SIVcpzANT has features that would class it as a naturally attenuated virus. The most that can be said is that SIVcpzANT is lacking any large-scale gene deletions or nonsense mutations, such as *nef* deletions found in some long-term non-progressor human patients [39,40]. Furthermore, it has been shown that SIVcpzANT *nef* cannot downregulate CD3, as with other SIVcpz isolates [11].

While there is no evidence of an AIDS like disease in these captive SIVcpz infected animals, the results here are not entirely contradictory with the findings in wild chimpanzees. Firstly, it bears restating that the known causes of death of the wild SIVcpz infected animals, trauma and massive parasite infection, are unlikely to occur in captive animals. However, as in wild chimpanzees, SIVcpz infection of these captive animals was associated with a loss of CD4+ T-cells. Furthermore, SIVcpz infection was associated with changes to the secondary lymphoid structure and increased collagen deposition in the spleen, as in wild chimpanzees.

Given that CD4+ T-cell loss is thought to be linked to immune activation in pathogenic infections, the relatively low levels of chronic immune activation seen in these captive animals most likely explains why CD4+ T-cell loss is only a gradual process. Furthermore, it could be suggested that additional sources of immune activation in the wild environment may lead to more rapid loss of CD4+ T-cells in wild chimpanzees.

In addition to the finding that wild SIVcpz infected chimpanzees suffer from increased mortality, one case of an AIDS like disease has been reported [25], and three more orphan SIVcpz infected chimpanzees have been reported to die rapidly following rescue to African primate centres or sanctuaries [41–43]. While the rapid deaths of some of these orphan chimpanzees was not proposed to be AIDS related at the time of the reports, the causes of death are notable in light of Keele *et al*; subacute pneumonia, parasite infection and severe diarrhea. It could be proposed that such African primate sanctuararies/centres are in many respects intermediate between the environment that the chimpanzees in this study were housed in, and the wild. While captive chimpanzees in African primate centres or sanctuaries have veterinary supervision, they have greater exposure to the natural environment, including the use of local fauna in both the food and enrichment materials. Exposure to infectious agents may be greater than in the environments of US/European primate research centres and sanctuaries—and if not greater, certainly different. The suggestion that these environments may be different in ways relevant to the outcome of SIVcpz infection is supported by the finding that soluble sCD14 levels are significantly different in animals housed in the US/European primate centres compared to animals housed at the CIRMF, Gabon. sCD14 is produced by a number of cell types in response to the bacterial cell wall molecule lipopolysaccharide (LPS), and thus elevated levels of sCD14 may indicate greater exposure to LPS. sCD14 has been used as a surrogate marker for integrity of the gut mucosal barrier, which is lost during progression to AIDS in pathogenic infections, and levels of soluble CD14 in HIV-1 infected humans predicts rate of disease progression [44,45]. Notably, pig-tailed macaques infected with SIV/SHIV are a model for AIDS, with generally rapid disease progression, and susceptibility to AIDS from a number of viruses that are not able to cause disease in rhesus macaques. It has been demonstrated that pre-infection, the gut mucosal barrier of these animals is frequently compromised, and these animals have higher levels of immune activation pre-infection than rhesus macaques [46]. Crucially, the level of bacterial translocation (as measured by levels of plasma LPS-binding protein) pre-infection is predictive of the rate of disease progression post infection [47]. Thus it seems possible that the higher levels of sCD14 in chimpanzees held in an African primate centre may be partly responsible for the apparent increased susceptibility to disease progression following SIV infection in wild and captive chimpanzees in Africa.

An additional finding that may explain the increased mortality found in wild African chimpanzees, but not these captive animals infected with SIVcpz, is the trend observed here for SIVcpz infection to be associated with anti-platelet antibodies and thrombocytopenia. Thromobocytopenia is a common complication of HIV-1 infected humans, occurring in 10–30% of HIV-1 infected patients [48,49]. The development of anti-GPIIIa antibodies (shown here to have the most compelling relationship between SIVcpz infection and thrombocytopenia in the chimpanzee cohort) has been shown to be specifically associated with patients developing HIV-1 associated immune thrombocytopenia [50]. It has previously been reported that molecular mimicry between HIV-1 gp120 and human gpIIIa leads to the development of this antibody in humans [51]. Identification of similar mimicry between SIVcpz gp120 and chimpanzee platelet proteins was beyond the scope of this study. Interestingly, chimpanzees infected with HIV-1 have also been documented as developing thrombocytopenia, in the presence of high levels of anti gpIIIa antibodies [52].

While under high levels of veterinary care, such as is found in primate centres and sanctuaries, the outcome of chronic thrombocytopenia is less likely to be severe than in the wild. Indeed, while Ch-No frequently suffers from nose-bleeds, the chronic thrombocytopenia from which this animal has suffered for over 15 years has yet to cause any life-threatening complications. In contrast, the chimpanzees in the Gombe national park live under more physically and environmentally demanding conditions. Clearly, a chronic thrombocytopenia is more likely to be life-threatening where aggressive encounters, high parasitic burdens, stress and trauma are more likely. Indeed, it can be questioned how long Ch-No, which has suffered from a profound thrombocytopenia from age 9 years, would have survived in the wild.

The question as to if the wild environment affects the outcome of SIV infection in other African primate species has recently been addressed by ourselves and others. Ma *et al*’s studies of wild African green monkeys [53,54], of exceptional scope and scale, found very few perturbations of the immune system in SIVagm infected monkeys. By contrast, our own study of naturally SIVmnd-1 infected mandrills, held in a ‘semi-wild’ environment, revealed significant loss of memory CD4+ T-cells, and significantly increased immune activation in infected animals [34]. These features of SIVmnd-1 infection were not found in captive, experimentally infected mandrills [55], suggesting that the wild environment may indeed alter the outcome of SIV infection.

While this study is limited by the relatively small number of SIVcpz infected chimpanzees and incomplete sample availability, it should be noted that future experiments involving SIVcpz infection of chimpanzees are extremely unlikely, due to changes in ethical and legal limitations placed on invasive research on chimpanzees in the period since these experiments were instigated. Thus, this work, based on retrospective analysis of stored samples and follow up of animals involved in earlier experiments provides the most thorough investigation to date, and for the foreseeable future, of the effect of SIVcpz infection on the immune system of captive chimpanzees.

To conclude, the history of SIVcpz infection of captive chimpanzees shares a number of features of both pathogenic and non-pathogenic infection. In common with the non-pathogenic SIV infection of well-studied African primates, the SIVcpz infected chimpanzees studied here rapidly control immune activation (when measured in the peripheral blood), and they appear to maintain the gut mucosal barrier. However, SIVcpz infection is associated with loss of CD4+ T-cells, and significant immune activation in the lymph node, and with changes in the structure of the secondary lymphoid environment. In the captive environment, these effects are insufficient to cause an overt AIDS-like disease, but do distinguish SIVcpz infection of chimpanzees from the SIV infections of other African primates. Thus, the virus-host relationship in SIVcpz infected chimpanzees appears to be intermediate between that found in well described ‘natural hosts’ and in species in which SIV/HIV infection leads to disease. Damage to the immune system in this cohort of captive SIVcpz infected chimpanzees was insufficient to lead to AIDS-like disease, but nevertheless, significant disruptions to the immune system were found, perhaps explaining why SIVcpz infected animals in the wild suffer an adverse outcome.

## Materials and Methods

### Animals

Experimental infection protocols have been described previously [16]. Animals Ch-No and Ch-Ni were housed at the BPRC, Netherlands, until the death of Ch-Ni in 2005, and the transfer of Ch-No to the Rescue Centre for Exotic Animals ‘Stichting AAP’. Animals X062, X310, X284, X176 and X115 were housed at the Texas Biomedical Research Institute. X115 was subsequently transferred to ‘Chimp Haven’ in 2006. Cam155 has been described previously, and was housed at the Ape Action Africa (AAA) sanctuary, Yaoundé, Cameroon at the time of sampling. Data from uninfected and HIV-1 infected animals were obtained from sexually mature chimpanzees (over age 7 years). HIV-1 infected chimpanzees were all infected for greater than five years at the date of data used.

### Ethics statement

Experimental infection of animals has been reported previously and was approved by the TBRI (formerly Southwest Foundation for Biomedical Research, SFBR, Animal Welfare Assurance Number A3082-01) IACUC, with the protocol assigned number 130-PT-4. Additional blood draws from animals held at Texas Biomedical were approved under protocol numbers 181-PT-3 and 825-PT-0. Experimental infection of Ch-Ni was approved by the IACUC of the BPRC, protocol 914a (DEC-004). Blood draws from uninfected and HIV-1 infected animals at the BPRC were approved under protocols 84-6A and 101–7 (DEC-029). Data was also acquired from HIV-1 and SIVcpz infected animals held at Chimp Haven, Louisiana, during an annual health check. When animals were anaesthetised and blood was already to be drawn for veterinary control purposes, a small volume of additional blood was taken to carry out flow cytometric and viral load analysis. Results were returned to veterinary staff of Chimp Haven to aid in the care of these animals. An additional blood draw for this purpose was approved by the ethical review board of Chimp Haven and assigned protocol number 2010–02. The use of residual blood drawn from Cam155, AAA, Cameroon, and uninfected chimpanzees at the CIRMF Gabon, taken for veterinary purposes during routine health checks, and remaining after these assays were carried out, was approved by the Ethical committee of the Department of Veterinary Medicine, University of Cambridge, with the approved protocol logged as CR90. At Stichting AAP, Ch-No was sedated annually in order to monitor his health, with focus on his thrombocytopenia and viral status. A small amount of blood was collected extra for cytometric and viral load analysis. This was performed under supervision of AAP’s Board of Experts and was agreed in a covenant between the Dutch government, the BPRC and Stichting AAP in June 2003.

Housing of animals in all centres complied with the Guide for the Care and Use of Laboratory Animals (TBRI) or The European Council Directive 86/609/EEC (BPRC, CIRMF, AAP, AAA). Animals in all centres were housed in spacious cages or open air ‘corrals’. Animals at the BPRC and TBRI and AAP were provided with commercial food pellets supplemented with appropriate treats, while animals at the CIRMF were fed with various Gabonese fruits and vegetables, a “home-made” protein complement cake, and other snacks between meals. In all cases, drinking water was provided *ad libitum*. In all cases, environmental enrichment was in all centres through swings, hammocks, raised platforms within the enclosures, and through the addition of supplementary toys, which were changed regularly. The housing conditions at the BPRC and AAP have also been described extensively previously [56]. All blood draws were taken under anaesthesia, ketamine/HCl, 10 mg/(kg body weight), and all efforts were made to minimise animal suffering. No chimpanzees were sacrificed for this study, and all post-mortem materials were recovered after death from other causes.

### Determination of lentiviral load

QC-PCR analysis of viral loads was carried out as previously described [57]. A *pol* based qRT-PCR assay was designed to replace this assay based on *pol* clones derived from the plasma of Ch-No, generated by nested PCR, along with previously published SIVcpz sequences. A lack of polymorphism in the selected region was confirmed by analysis of sequences generated by Illumina sequencing of RNA sequences from plasma at week two p.i. from Ch-Ni. The primers selected were GGTAATGGCAGATGAGACAGG (forward) and TGGATTCCACTACTCCTTGAC (reverse), with the probe JOE-GGCCATCTGCTGGCTAATTTTAACAGGAA-BHQ1, where JOE is the fluorochrome and BHQ1 is the quencher (Black Hole Quencher 1). Comparison of samples measured with both the QC-PCR and qRT-PCR assays showed reasonable agreement (S3 Fig).

To carry out the assay, RNA was extracted from 200 μl of plasma using the Roche High Pure Viral RNA kit as per manufacturer protocol. Extracted RNA (in a total of 50 μl) was supplemented with RNAsin Plus RNase inhibitor (Promega) at one unit per μl and immediately frozen at -80°C. Frozen RNA was thawed on ice, and 10 μl of RNA was added to 10ul real-time reaction mastermix (Taqman Fast Virus One-step real-time PCR Mastermix, including primers and probe). Serial dilution of a plasmid bearing the pol region of SIVcpzANT was used as a standard. The real-time assay was carried out on a Rotorgene 6000 (Qiagen) using the following protocol; 5 min at 50°C (for reverse transcription), 20 s at 90°C (to inactivate the reverse transcriptase and for initial denaturation), followed by 40 cycles of 95°C for 3 s and 60°C for 30 s. This assay did not detect HIV-1, as confirmed by using plasmids containing the *pol* of HIV-1 IIIB and SF2.

HIV-1 viral loads were determined using the QC-PCR assay [57], or a taqman assay as described previously [58]. Viral loads of HIV-1 infected chimpanzees were generally low. Of the 29 HIV-1 non-progressor animals included in Fig 1, on the date of data shown, 18 had viral loads <100 copies/ml, two had viral loads between 100 and 1000 copies/ml, and three had viral loads between 1000 and 5000 copies/ml. Six animals did not have their HIV-1 viral loads determined on the same date, but other measurements for these animals fell in the same range (<100 to 5x10^4^ copies/ml). As previously described, in SIVcpz infected animals that were infected with HIV-1 at the point of SIVcpz infection, HIV-1 viral loads were detectable only in X310 and X115 at the point of infection. HIV-1 viral loads of X310 were consistently between <200–500 copies/ml until the point of death. HIV-1 viral loads of X115 were also between 2x10^2^ and 5x10^3^ during the first year of SIVcpz infection, but have been <100 copies/ml in every sample taken subsequently.

### Analysis of plasma markers and auto-immune antibodies

Plasma samples were stored at -80°C until use. Commercial ELISAs were used to determine the concentration of soluble markers of immune activation or auto-immune antibodies. The suppliers of these assay are listed in parentheses; sCD14 (Abcam), sTRAIL (Abcam), β2 microglobulin (IBL international), Neopterin (IBL neopterin), PAKAUTO assay for anti-platelet antibodies (Gen-Probe). All assays were used according to manufacturer protocol, using frozen plasma, with the exception that packed platelet samples were unavailable for the PAKAUTO assay, and therefore only free antibodies in plasma could be measured. All samples were assayed together at the University of Cambridge after shipment on dry ice from the respective centres involved.

### Flow cytometry

Flow cytometry on fresh samples was carried out on whole blood. 100 μl of whole blood was incubated with the antibody mixture for 15 min at room temperature in the dark, in round bottomed polystyrene tubes (BD Biosciences). The sample was treated with FACS lysing solution (BD Biosciences) for 10 min in the dark to lyse erythrocytes, centrifuged, and washed with 2 ml of PBS with 1% bovine serum albumin. Finally, the cells were fixed with 2% paraformaldehyde in PBS overnight and analyzed. Due to the long period of time of this study and the different centres involved, analysis was carried out on number of different flow cytometers (FACSscan, FACScalibur, LSR-II or FACSAria, all manufactured by BD Biosciences).

Staining of cryopreserved samples was carried out to acquire data on Ki-67 expression in retrospective samples as indicated. At the time of sampling, PBMCs were isolated over a Ficoll-Pacque gradient (GE healthcare) and frozen in a medium of 90% foetal calf serum, 10% Dimethyl sulfoxide. An initial gradual temperature decline was achieved using a ‘Mr Frosty’ (Nalgene) container with isopropanol and a -80°C freezer. Once frozen, samples were stored in a liquid nitrogen container. Samples were thawed quickly at 37°C, washed once in warm RPMI-1640 including benzonaze (50 units/ml), and incubated for 1 hour at 1x10^6^ cells/ml in RPMI-1640 with 10% FCS for one hour at 37°C, to allow restoration of cell membrane integrity. 1 ml (1x10^6^) cells were then transferred to round bottomed polypropylene tubes (BD Biosciences), centrifuged, and resuspended in 100 μl of antibody mixture in PBS, including a viability marker to allow the exclusion of dead cells after acquisition.

Antibodies against the following markers were used, with the clone name in parentheses, all supplied by BD; CD3ε (SP34.2), CD4 (SK3), CD8 (SK1), CD16 (3G8), CD20 (L27), CD69 (FN50), MHC-II (anti-HLA-DR, L243), Ki-67 (B56).

### Transcriptomics analysis

PBMC pellets containing 5x10^6^ PBMCs were snap frozen at the indicated time-point. Pellets were resuspended in 0.5 ml Qiazol (Qiagen) and incubated for 5 m at room temperature. RNA extraction was then performed using the Qiagen RNeasy Lipid Tissue mini kit, as per manufacturer instructions, including on-column DNA digest. RNAsin Plus (Promega) RNA inhibitor was added to eluted RNA, and RNA samples were stored at -80°C at this point. As RNA yield was low, the Ovation RNA-Seq FFPE kit (Nugen) was used, as per manufactures instructions to provide sufficient cDNA for the standard Illumina RNA-seq protocol, with 100bp paired reads, carried out by the TGAC sequencing centre, Norwich. As the RNA yield was limiting, some samples were excluded at this point, and for almost all samples, all purified RNA was required for the amplification and subsequent sequencing processes. qRT-PCR validation of this data-set could therefore not be carried out. Analysis of sequencing data was carried out using the CLC genomics platform. Sequence data received from the TGAC (8–70 million reads per sample) was trimmed to remove low complexity and low quality reads. Subsequently, reads were mapped to the chimpanzee annotated genome (Ensemble CHIMP 2.1.4.74), resulting in approximately 70% of reads mapping uniquely to the chimpanzee genome, with each read in a pair counting once, to allow inclusion of reads mapping as a broken pair, and pairs broken during the trimming process. Total numbers of unique gene reads were normalised to the number of mapped reads per sample, and this number was used for intergroup comparisons. Heatmaps were generated using the gplot package, executed in R [59]. Genes were selected for inclusion for figures on the basis that reads were detected in all available samples and had a more than two-fold difference in any group compared to the control group. Heatmaps shown in the main body figures include genes relevant to the post-translational measurements within the same figures. S4 Fig shows genes that were more than twofold up or down regulated in any group compared to the uninfected control, that were more than two-fold upregulated in a human *in vitro* T-cell activation study at 24, 48 or 72 hours post activation [60]. S5 Fig shows genes that were more than twofold up or down regulated in any group compared to the uninfected controls, that appear more in more than 10 datasets when queried on the human interferome, version 2.01 [61].

Open access to the transcriptomics reads is available from the ENA under accession ERP009138. S7 Table gives the number of reads per gene in each sample, per million reads.

### Immunohistochemistry

Immunohistological examination was performed on sections of formalin fixed, paraffin embedded tissues. Unfortunately, as animal X310 died during the night, and was not discovered until the morning, tissues from this animal were subject to autolysis sufficient to render them unusable for analysis. Following dewaxing, heat-induced epitope-retrieval was carried out using the PT link module (Dako), before they were stained using the EnVision FLEX Kit (Dako) in combination with a Dako Autostainer according to the manufacturer protocol. Primary antibodies against the following molecules were used, with the clone name, manufacturer and dilution factor in parentheses; CD3 (F7.2.38, Dako, 1:300), Ki-67 (M1B-1, Dako, 1:150), MX1 (polyclonal #95926, Abcam, 1:500), Collagen-1 (Rabbit polyclonal, ab34710, Abcam, 1:500). Collagen-I staining of samples from rhesus macaques were excluded from the analysis, as similar intensity of staining could not be achieved compared to chimpanzees.

### Quantitative tissue analysis

Slides were scanned using a Nanozoomer 2.0 brightfield whole-slide scanner (Hamamatsu) at 40x magnification. For analysis of lymphoid hyperplasia, the outline of follicles was traced manually on scans of H&E stained slides, using ImageScope (Version 17, Aperio), which outputs the area of the object outlined. Quantitative analysis of Mx1 and Collagen-I was also carried out on ImageScope. T-cell zones were traced on CD3+ slides (stained on consecutively cut slides) and superimposed onto Mx1 or Collagen-1 slide scans. The proportion of the area staining for these markers was then determined using the positive pixel count tool within ImageScope.

To quantify the number of Ki-67+ cells within the T-cell zone a slightly different approach was used. 0.01 mm^2^ areas were captured from individual T-cell zones in the Ki-67 stained slide (based on the CD3+ stained slide) at 40x magnification. A selection of these images from several different animals was then used to train an image classifier in Ilastik (version 0.5 [62]). Ilastik is a machine learning programme. Areas representing Ki-67+ and Ki-67- cells, in addition to the background are manually specified by tracing onto the images selected. Ilastik then uses this information to generate a classifier that can be used to define these three groups in any image. Cellprofiler (version 2.0 [63]) was then used to count the number of Ki-67+ cells. A cellprofiler pipeline was constructed to import all Ki-67+ images in turn, and use the classifier defined by Ilastik to define Ki-67+ areas. The object counting module was then used to define individual Ki-67+ cells based on size and shape parameters determined empirically. This allowed for objective counting of cells even where positive cells were closely clustered.

### Statistical analysis

General linear models were used to determine the effect of HIV-1 and SIVcpz infection, in addition to age and gender, after variables were transformed where necessary to produce normally distributed data. This analysis was carried out using the R platform [59]. Where data could not be normalised satisfactorily, or where the number of values was too low to allow confidence that data was appropriately distributed, a Kruskall-Wallis test with Dunn’s multiple comparison was carried out using Prism 5.0 (Graphpad).

## Supporting Information

S1 Fig Longitudinal measurements of viral load and CD4+ T-cell count in Ch-No. Viral load was measured using a quantitative competitive PCR (QC-PCR) or a reverse transcriptase, real-time PCR (qRT-PCR) (see S3 Fig). Broken black line indicates limit of detection of the viral load assays (250 copies/ml).
(PDF)

S2 Fig Longitudinal CD4+ T-cell counts and viral loads in the experimentally SIVcpz infected chimpanzees. Absolute peripheral blood CD4+ T-cell counts are indicated in blue, while viral loads (determined with the qRT-PCR assay are displayed in red). Time points in the first year of infection for which viral load data were available are indicated on the axis in red numerals, as differences in availability of samples results in apparent differences in early dynamics of the viral load. Broken blue and red lines indicate long periods between data points. The broken black line indicates the limit of detection of the viral load assay (250 copies/ml).
(PDF)

S3 Fig Comparison of the QC-PCR and qRT-PCR viral load assays. Viral load was measured using a quantitative competitive PCR (QC-PCR) or a reverse transcriptase, real-time PCR (qRT-PCR) as described in the methods section. (A) Viral loads determined on the same samples with both assays showed a good correlation in samples from Ch-No, where the use of both methods was required due to lack of availability of samples to repeat measurements with the qRT-PCR assay.
(PDF)

S4 Fig T-cell activation associated gene expression in PBMCs. Transcription level expression of genes known to be upregulated in human *in vitro* activated T-cells that were or down regulated by at least two fold in any group compared to the control animals in the RNAseq cohort (n = 3).
(PDF)

S5 Fig Interferon inducible gene expression in PBMCs. Transcription level expression of known interferon inducible genes in PBMCs in the RNA-seq cohort that were up or down regulated by at least two fold in any group compared to the control animals (n = 3).
(PDF)

S6 Fig Analysis of Ki-67 expression in the T-cell zones of SIVcpz infected chimpanzees. (A) Representative images of anti-Ki-67 staining in the T-cell zones of a lymph node of an uninfected chimpanzee, and of Ch-Ni at week 2 and 12 post infection, and at necropsy. (B) Number of Ki-67+ cells in the T-cell zone of lymph nodes over the course of experimental infection and (C) in chronic SIVcpz infected chimpanzees, SIVmac infected rhesus macaques and control animals. Horizontal black bars indicate the mean value in each group.
(PDF)

S1 Table Summary of the age and gender attributes for SIVcpz and control chimpanzee groups for CD4+ T-cell analysis (Fig 1). (XLS)

S2 Table Details and coefficients of statistical analysis used in (Fig 1). (XLS)

S3 Table Summary of the age and gender attributes for SIVcpz and control chimpanzee groups for T-cell activation analysis (Fig 2b). (XLS)

S4 Table Details and coefficients of statistical analysis used in T-cell activation analysis (Fig 2b). (XLS)

S5 Table Summary of the age and gender attributes for SIVcpz and control chimpanzee groups for transcriptomics analysis. (XLS)

S6 Table Summary of the age and gender attributes for SIVcpz and control chimpanzee groups for soluble marker analysis (Fig 3). (XLS)

S7 Table Details of samples used for histological and immunohistochemical analyses. (XLS)

S8 Table Transcriptomics data analysis values. Values are total reads mapping to each gene, normalised to the number of reads mapped in each sample. All time-points post-infection (or pre-infection, indicated by a negative value) are related to SIVcpz (not HIV-1) infection.
(XLS)
