# Supplementary figures and images for: Simian Immunodeficiency Virus Infection of Chimpanzees (Pan troglodytes) Shares Features of Both Pathogenic and Non-pathogenic Lentiviral Infections
Source: PLoS Pathog. 2015 Sep 11;11(9):e1005146. doi: 10.1371/journal.ppat.1005146 (PMC4567047; doi:10.1371/journal.ppat.1005146)

# Ch-No

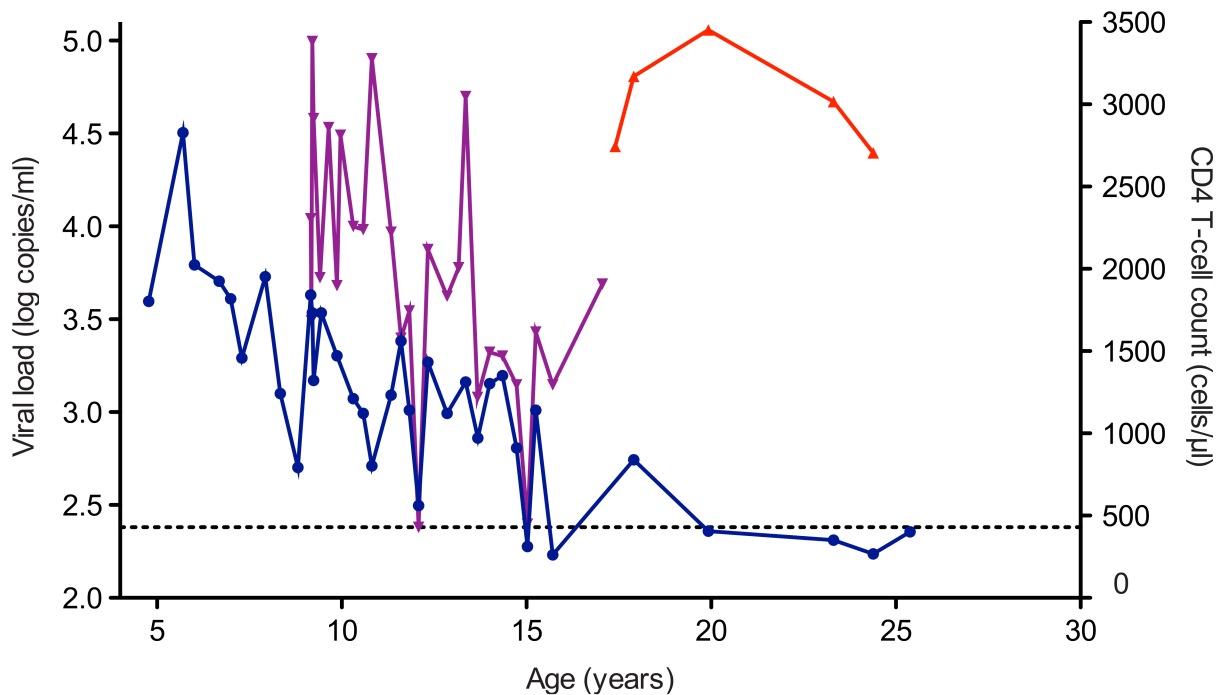

Supplement: S1 Fig — Viral load was measured using a quantitative competitive PCR (QC-PCR) or a reverse transcriptase, real-time PCR (qRT-PCR) (see S3 Fig). Broken black line indicates limit of detection of the viral load assays (250 copies/ml). (PDF) [file ppat.1005146.s001.pdf]

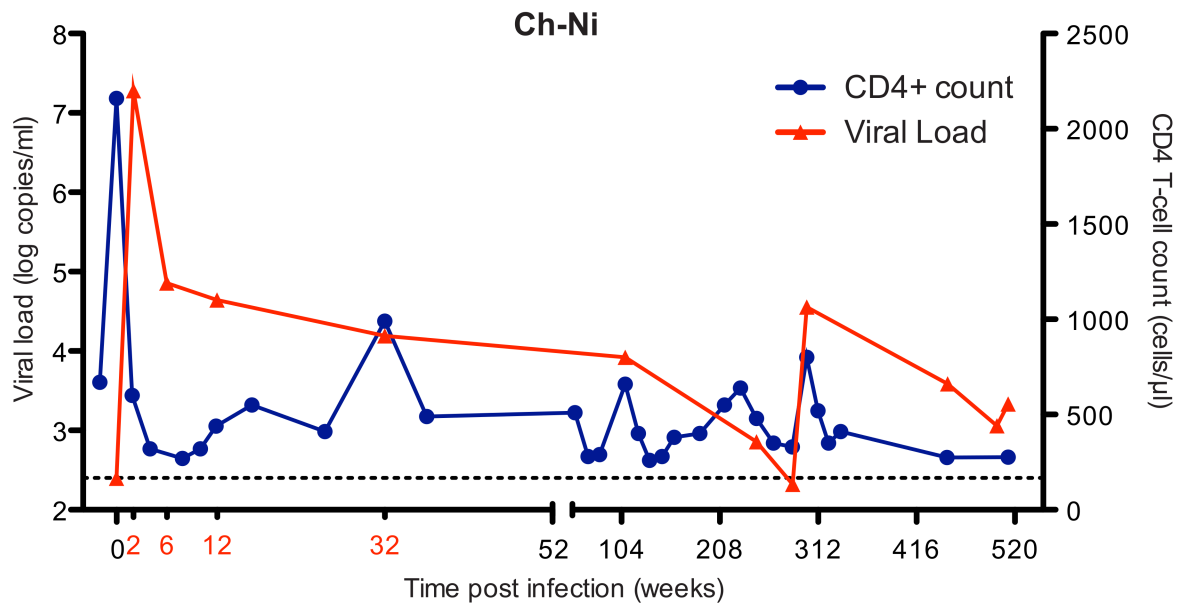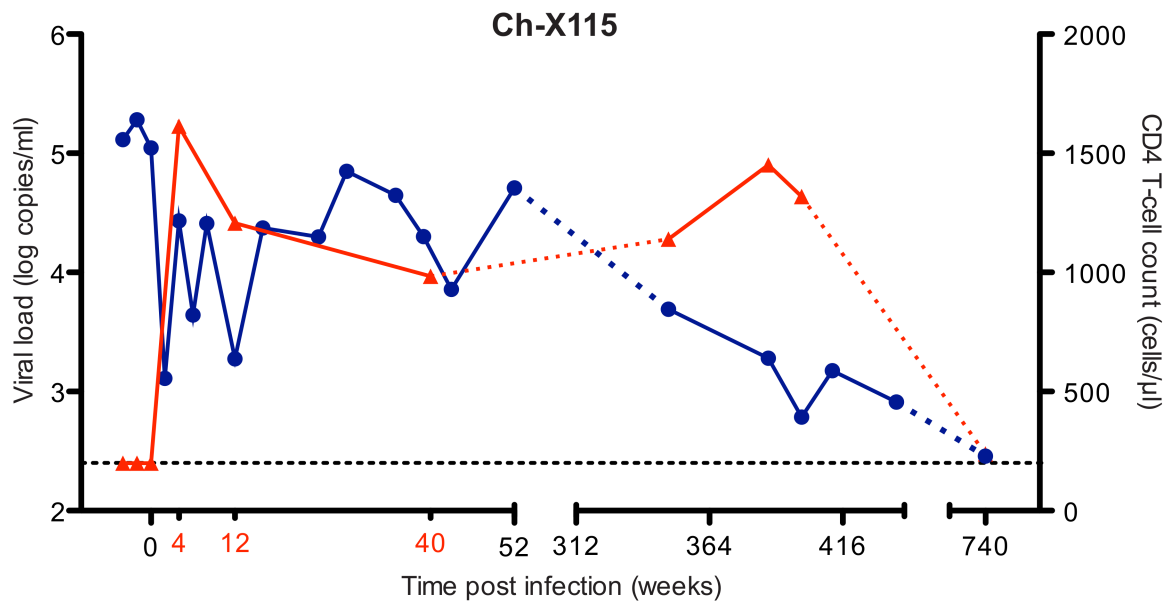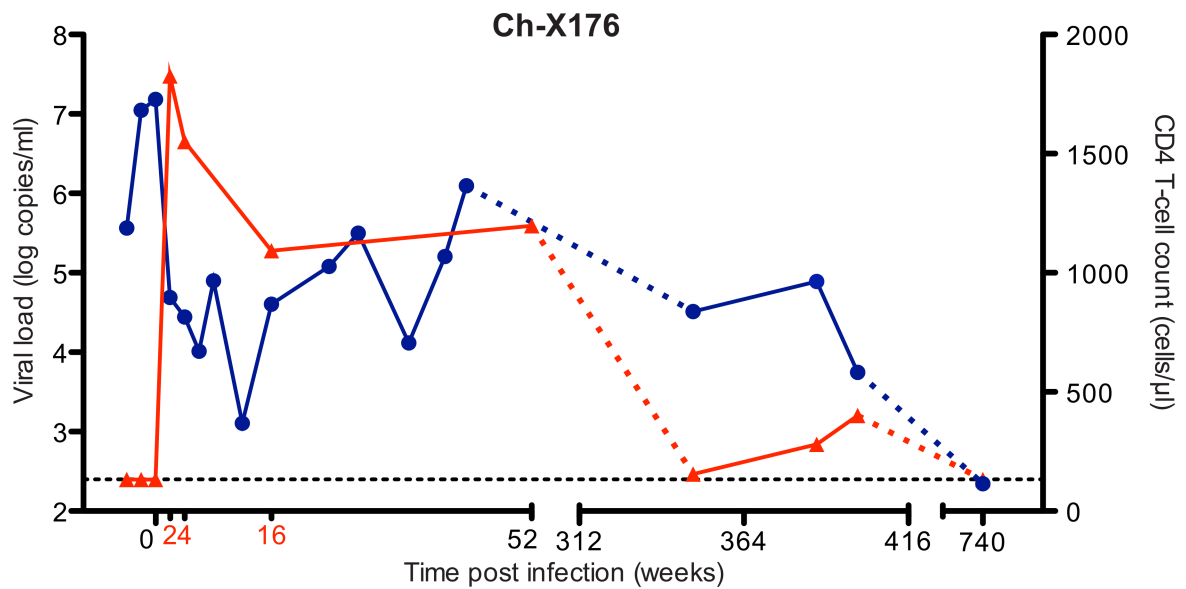

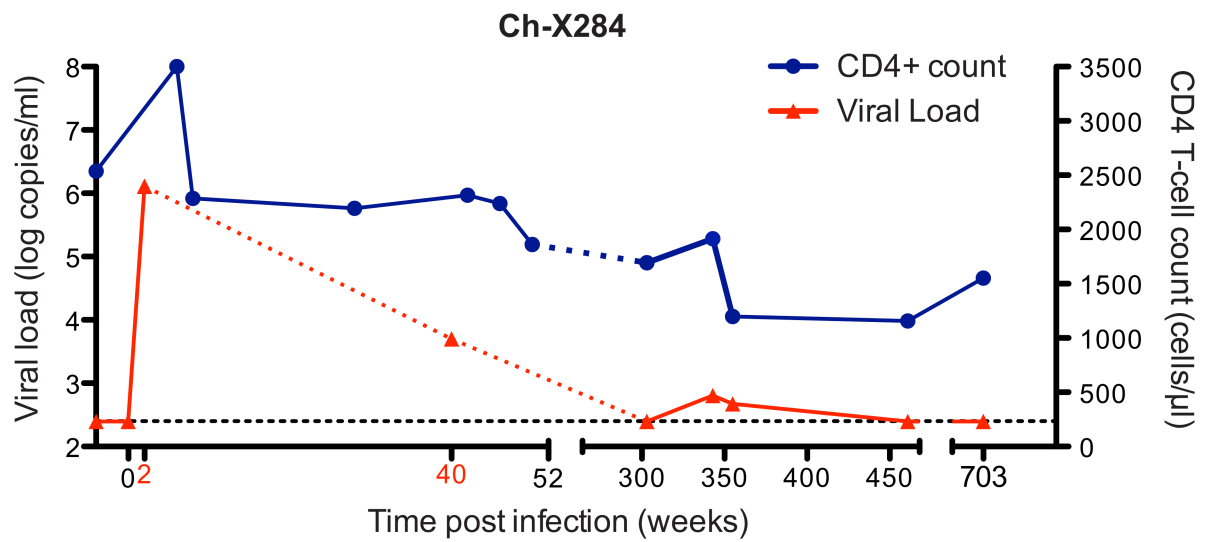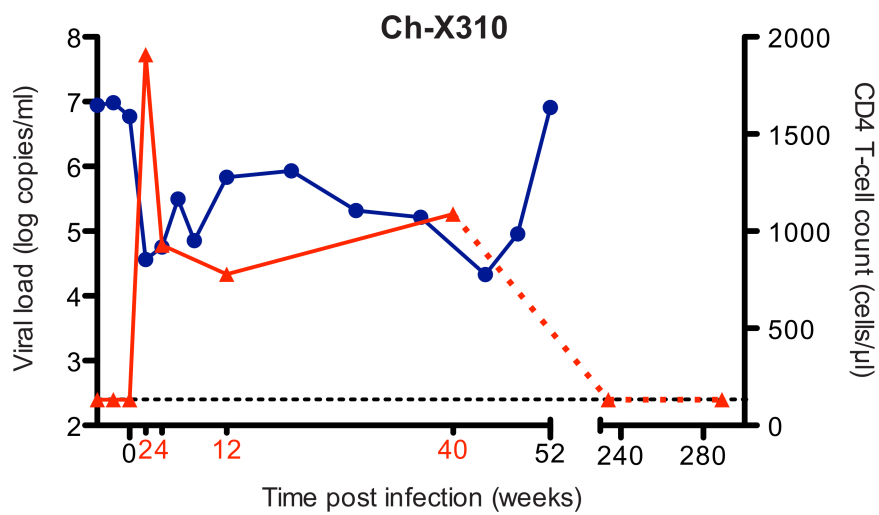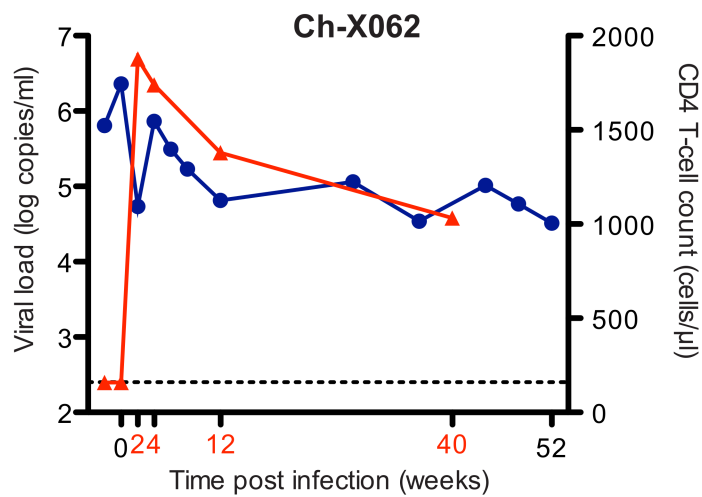

Supplement: S2 Fig — Absolute peripheral blood CD4+ T-cell counts are indicated in blue, while viral loads (determined with the qRT-PCR assay are displayed in red). Time points in the first year of infection for which viral load data were available are indicated on the axis in red numerals, as differences in availability of samples results in apparent differences in early dynamics of the viral load. Broken blue and red lines indicate long periods between data points. The broken black line indicates the limit of detection of the viral load assay (250 copies/ml). (PDF) [file ppat.1005146.s002.pdf]

**A**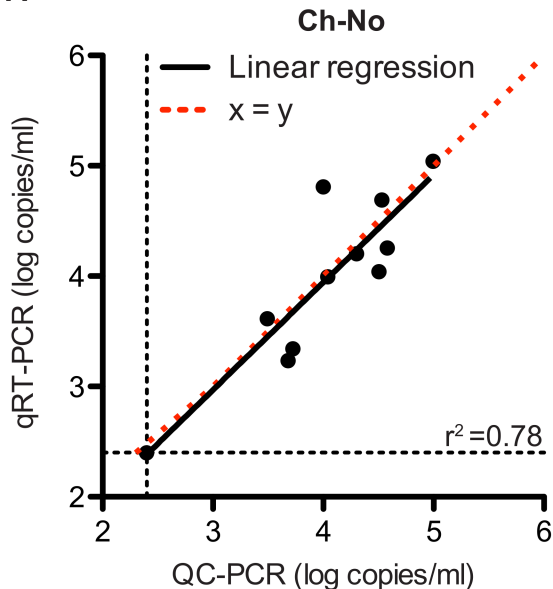

Supplement: S3 Fig — Viral load was measured using a quantitative competitive PCR (QC-PCR) or a reverse transcriptase, real-time PCR (qRT-PCR) as described in the methods section. (A) Viral loads determined on the same samples with both assays showed a good correlation in samples from Ch-No, where the use of both methods was required due to lack of availability of samples to repeat measurements with the qRT-PCR assay. (PDF) [file ppat.1005146.s003.pdf]

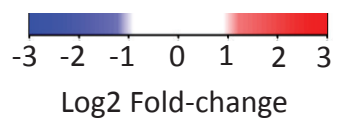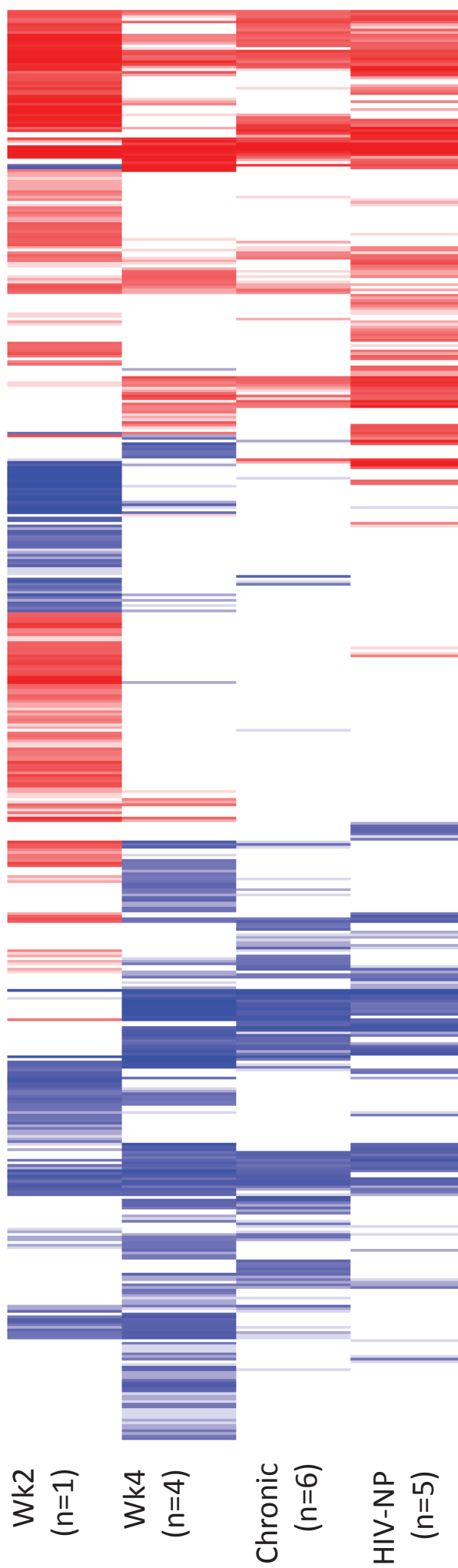

Supplement: S4 Fig — Transcription level expression of genes known to be upregulated in human in vitro activated T-cells that were or down regulated by at least two fold in any group compared to the control animals in the RNAseq cohort (n = 3). (PDF) [file ppat.1005146.s004.pdf]

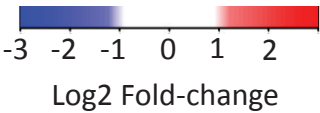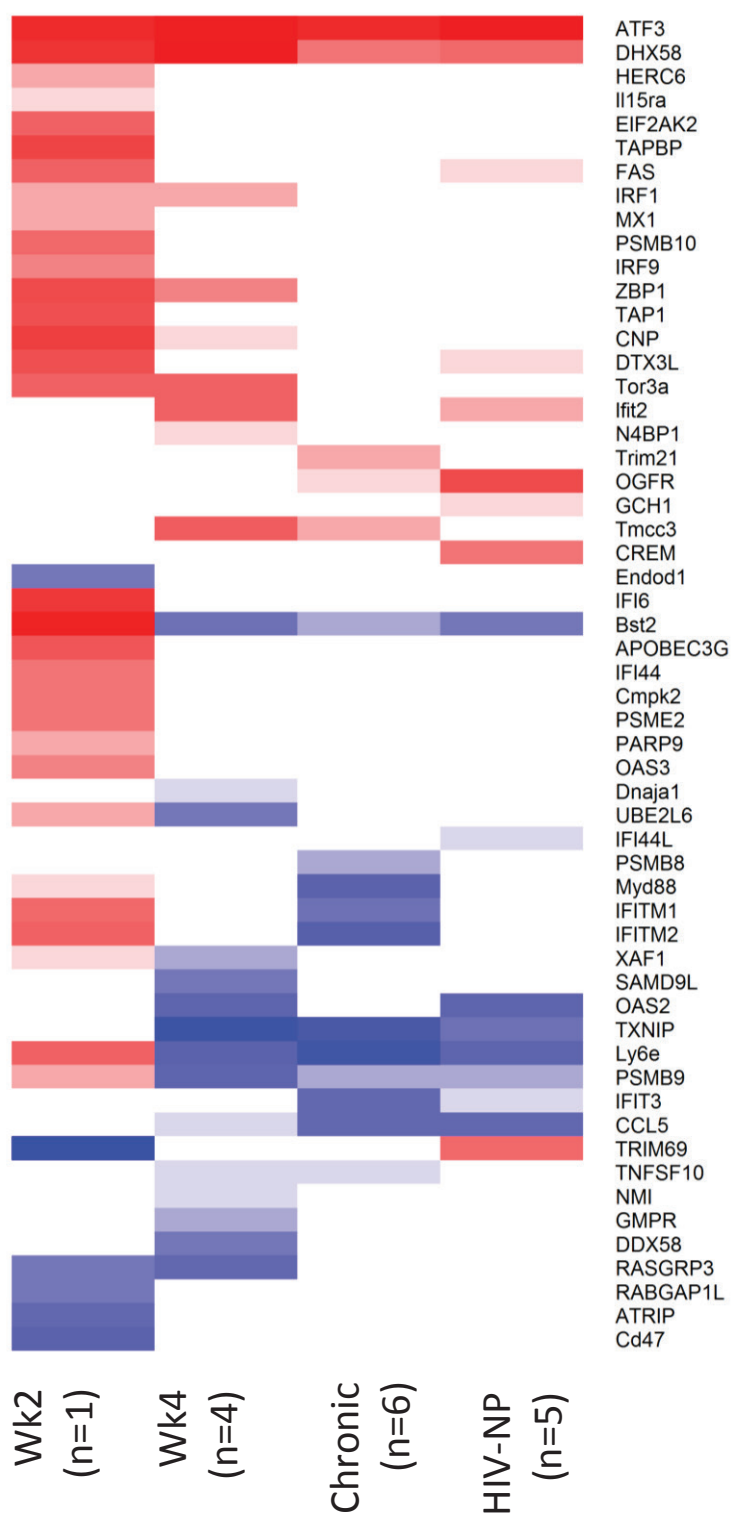

Supplement: S5 Fig — Transcription level expression of known interferon inducible genes in PBMCs in the RNA-seq cohort that were up or down regulated by at least two fold in any group compared to the control animals (n = 3). (PDF) [file ppat.1005146.s005.pdf]

**A**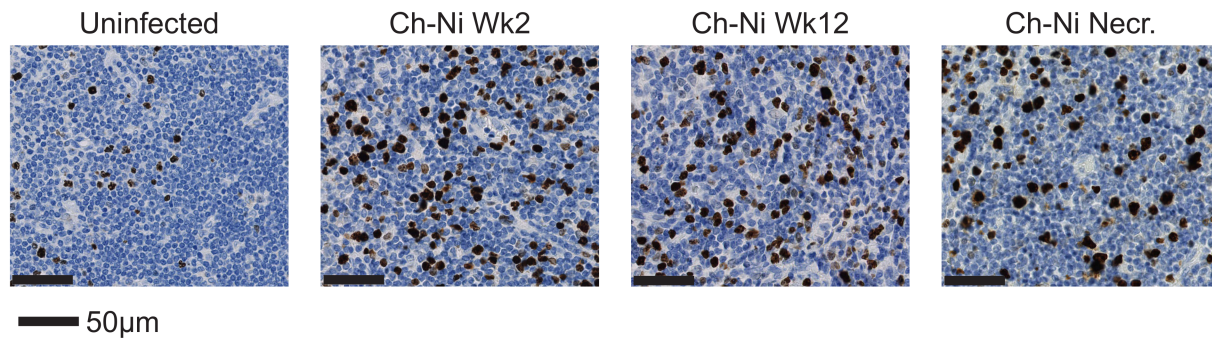**B**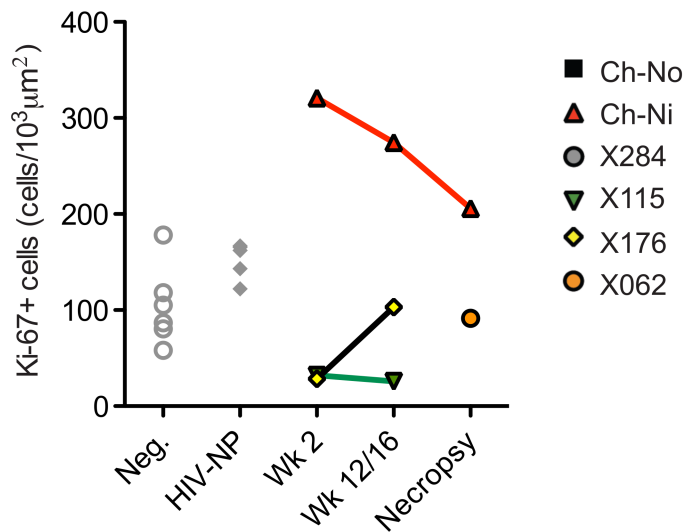**C**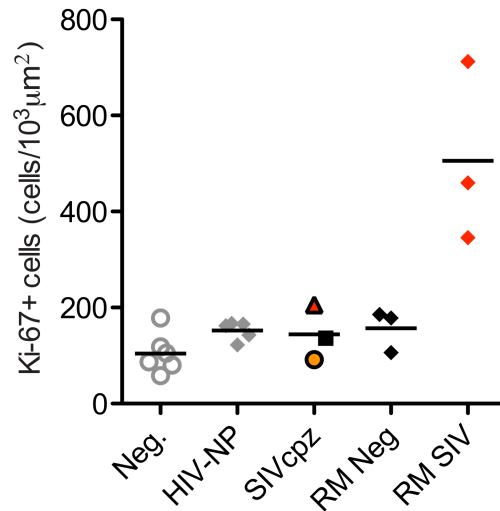

Supplement: S6 Fig — (A) Representative images of anti-Ki-67 staining in the T-cell zones of a lymph node of an uninfected chimpanzee, and of Ch-Ni at week 2 and 12 post infection, and at necropsy. (B) Number of Ki-67+ cells in the T-cell zone of lymph nodes over the course of experimental infection and (C) in chronic SIVcpz infected chimpanzees, SIVmac infected rhesus macaques and control animals. Horizontal black bars indicate the mean value in each group. (PDF) [file ppat.1005146.s006.pdf]
